# Myrosinase isogenes in wasabi (*Wasabia japonica* Matsum) and their putative roles in glucosinolate metabolism

**DOI:** 10.1186/s12870-024-05057-w

**Published:** 2024-05-01

**Authors:** To Quyen Truong, Yun Ji Park, Je-Seung Jeon, Jaeyoung Choi, Song Yi Koo, Yeong Bin Choi, Phuong Kim Huynh, Jinyoung Moon, Sang Min Kim

**Affiliations:** 1 Division of Bio-Medical Science & Technology, Korea Institute of Science and Technology (KIST), University of Science and Technology, Seoul, 02792 Republic of Korea; 2 Smart Farm Research Center, KIST Gangneung Institute of Natural Products, Gangneung, 25451 Republic of Korea; 3 Department of Herbal Crop Research, National Institute of Horticultural and Herbal Science, RDA, Eumseong, 27709 Republic of Korea; 4 Department of Oriental Medicine Biotechnology, College of Life Sciences, Kyung Hee University, Yongin, 17104 Republic of Korea; 5 Natural Product Informatics Research Center, KIST Gangneung Institute of Natural Products, Gangneung, 25451 Republic of Korea

**Keywords:** Glucosinolates, Glucosinolate hydrolysis products, GSL-MYR defense system, Myrosinase, *Wasabia japonica*, Abiotic stress, Methyl jasmonate

## Abstract

**Background:**

Wasabi, a Brassicaceae member, is well-known for its unique pungent and hot flavor which is produced from glucosinolate (GSL) degradation. Myrosinase (MYR) is a principle enzyme catalyzing the primary conversion of GSLs to GSL hydrolysis products (GHPs) which is responsible for plant defense system and food quality. Due to the limited information in relation to MYRs present in wasabi (*Wasabia japonica* M.), this study aimed to identify the *MYR* isogenes in *W. japonica* and analyze their roles in relation to GSL metabolism.

**Results:**

In results, *WjMYRI-1* was abundantly expressed in all organs, whereas *WjMYRI-2* showed only trace expression levels. *WjMYRII* was highly expressed in the aboveground tissues. Interestingly, *WjMYRII* expression was significantly upregulated by certain abiotic factors, such as methyl jasmonate (more than 40-fold in petioles and 15-fold in leaves) and salt (tenfold in leaves). Young leaves and roots contained 97.89 and 91.17 µmol‧g^−1^ of GSL, whereas less GSL was produced in mature leaves and petioles (38.36 and 44.79 µmol‧g^−1^, respectively). Similar pattern was observed in the accumulation of GHPs in various plant organs. Notably, despite the non-significant changes in GSL production, abiotic factors treated samples enhanced significantly GHP content. Pearson’s correlation analysis revealed that *WjMYRI-1* expression significantly correlated with GSL accumulation and GHP formation, suggesting the primary role of *WjMYRI-1*-encoding putative protein in GSL degradation. In contrast, *WjMYRII* expression level showed no correlation with GSL or GHP content, suggesting another physiological role of *WjMYRII* in stress-induced response.

**Conclusions:**

In conclusions, three potential isogenes (*WjMYRI-1*, *WjMYRI-2*, and *WjMYRII*) encoding for different MYR isoforms in *W. japonica* were identified. Our results provided new insights related to MYR and GSL metabolism which are important for the implications of wasabi in agriculture, food and pharmaceutical industry. Particularly, *WjMYRI-1* may be primarily responsible for GSL degradation, whereas *WjMYRII* (clade II) may be involved in other regulatory pathways induced by abiotic factors.

**Supplementary Information:**

The online version contains supplementary material available at 10.1186/s12870-024-05057-w.

## Background

*Wasabia japonica* Matsum or *Eutreuma japonicum* (wasabi), also known as Japanese horseradish, is a perennial herb native to Japan. Wasabi belongs to the Brassicaceae family, which includes widely consumed vegetables, such as broccoli, cabbage, kale, radish, and turnip. Wasabi plant features a main taproot, heart-shaped green leaves, and long, thin petioles. Although the whole plant is edible, the rhizome derived from the stem is the most highly prized part. Wasabi cultivation has been expanded to other countries: Canada, China, New Zealand, and South Korea aside from Japan because of the increasing global demand for wasabi-derived food products.

Wasabi is a widely used condiment due to a unique pungent and hot flavor. Isothiocyanates are primarily responsible for the characteristic flavors of cruciferous species, including wasabi. Notably, isothiocyanates are not synthesized in plant tissues as secondary metabolites but as hydrolysis products of glucosinolate (GSL) precursors. GSL is a group of sulfur-rich secondary metabolites synthesized from amino acids, such as methionine, tryptophan, and phenylalanine. GSLs are characterized by a core structure of sulfated isothiocyanate conjugated to thioglucose and a side chain. To date, more than a hundred distinct GSLs have been identified and further categorized as aliphatic, indolic, and aromatic GSL.

Although GSL content indicates the nutritional value of cruciferous plants, most GSLs are inactive and do not exert pharmaceutical properties. Health benefits are mainly derived from the breakdown products of GSLs [1]. Isothiocyanates, indoles, thiocyanates, simple nitriles, epithionitriles, and oxazolidine-2-thiones are common hydrolytic products of GSL degradation. Isothiocyanates are considered the most desirable products because of their contribution to taste, flavor perception, and bioactivities [2]. A wide range of flavors and tastes associated with GSLs/isothiocyanates have been characterized. For example, sinigrin/allyl isothiocyanate is known for its bitter taste and pungent flavor, gluconapin/3-butenyl isothiocyanate is associated with a wasabi-like flavor, and glucoibervirin/ibervirin and glucoerucin/erucin exhibit a radish-like flavor [3]. Furthermore, isothiocyanates exhibit various pharmaceutical activities, including anticancer, antimicrobial, and anti-inflammatory [4]. For instance, gaseous allyl isothiocyanate can inhibit the growth of some common food spoilage bacteria and pathogenic fungi [5].

Myrosinases (MYRs), also known as *β-*thioglucoside glucohydrolases (EC 3.2.1.147), are endogenous enzymes coexisting in all GSL-containing Brassicaceae plants, including *Arabidopsis thaliana*, horseradish (*Armoracia rusticana*), black mustard (*Brassica juncea*), rapeseed (*Brassica napus*), broccoli (*Brassica oleracea*), radish (*Raphanus sativus*), white mustard (*Sinapis alba*), and wasabi (*W. japonica*) [6]. It can also be produced by some bacteria (*Citrobacter* sp.), fungi (*Aspergillus niger*), insects (*Brevicoryne brassicae*, *Plutella xylostella*), and even in the human intestinal microbiome [7, 8].

In the late 1990s, the structure of MYRs was first characterized in white mustard seeds. Since then, more than a hundred MYRs have been identified in various species [6]. In general, MYRs can be divided into typical and atypical forms. Typical MYRs can be subdivided into clade I, which includes the MA, MB, and MC subfamilies, and clade II. MYRs are responsible for breaking GSL molecules into D-glucose and unstable aglycones, which are then converted into various products, depending on other factors [9]. Isothiocyanates are the most common products of GSL degradation. However, the presence of epithiospecifiers and thiocyanate-forming proteins can cause the further conversion of isothiocyanates into thiocyanates, simple nitriles, and epithionitriles.

Upon tissue disruption, MYRs and GSLs are released from cellular compartments. MYRs then catalyze the conversion of GSLs into degraded compounds that can deter or attract generalist and specialist species [10]. For example, the production of simple nitriles in *A. thaliana* can affect the behavior and reproduction of the white butterfly *Pieris rapae* L. [11]. Noticeably, *B. rapa* with high MYR levels shows high resistance against the flea beetle *Phyllotreta cruciferae*, or *Phyllotreta xylostella* causes less damage to *B. juncea* lines with high MYR activities [12]. Therefore, MYR plays critical role in plant defense system and is also responsible for GSL turnover and determines food quality [9]. In addition, MYR is involved in plant growth and development, although the underlying mechanisms are unclear. Along with GSLs, exposure to different abiotic factors, such as drought, temperature, light, phytohormones, and salinity, can either increase or decrease MYR activity [13, 14].

MYRs exhibit various isoforms and are known to be organ and species specific. For instance, *A. thaliana* has six MYR isoforms: TGG1, TGG2, TGG3, TGG4, TGG5, and TGG6 [15]. *AtTGG1* and *AtTGG2* are present in the upper parts of *Arabidopsis* plant, whereas *AtTGG4* and *AtTGG5* are present only in roots. *AtTGG3* and *AtTGG6* are pseudogenes expressed in the stamen and petals. Similarly, *A. rusticana* has two MYR isoforms: Myr1 and Myr2. Two MYR isoforms are also found in *R. sativus*. In the 1970s, MYR was first purified from *W. japonica* [16]. In 2005, Saitoh et al. [17] isolated full-length cDNA encoding MYR from wasabi petiole; however, little information has been reported about MYR existing in wasabi since then. Especially, other *MYR* isogenes in *W. japonica* remain to be identified. Therefore, the present study aimed to identify different *MYR* isogenes in *W. japonica* and their putative roles in GSL metabolism.

In this study, de novo assembly of raw sequence reads from wasabi transcriptomes identified potential *MYR* sequences encoding for MYR isoforms in *W. japonica*. The expression of these isogenes was investigated in different mature organs of vegetative plants subjected to abiotic treatments. The production of GSLs and GSL hydrolysis products (GHPs) was determined, and their correlation with *MYR* expression was investigated. In the present study, the comparison of MYR clades I and II in wasabi was provided for the first time. Our results would provide a new insight on different MYRs in *W. japonica,* which are important for the implications of wasabi in agriculture, food and pharmaceutical industries.

## Results

### Identification of different *MYR* isogenes in *W. japonica*

According to the BLASTX’s result, seven different unigenes were detected as best hits for myrosinase sequences of *A. thaliana* (*AtTGG*). Among them, three query sequences encoding MYR isoforms in *W. japonica* were identified with full coding region (Table 1).

**Table 1:** Information of myrosinase isogenes identified in *Wasabia japonica* using de novo assembly of raw sequencing reads available in National Center for Biotechnology Information library

Gene name (Abbreviation in this study)	Unigene no	Accession no	Amino acid (aa)	Orthologs (Accession no.)	Organism	Identity of aa sequence
*Myrosinase isoform 1* (*WjMYRI-1*)	TRINITY_DN2813_c0_g1_i4	AB194903.1	545	*Myrosinase* (XM_006394786.2)	*Eutrema salsugineum*	86%
				*TGG2-13* (MW413532.1)	*Isatis indigotica*	82%
				*Myrosinase* (XM_048767923.1)	*Brassica napus*	80%
*Myrosinase isoform 2* (*WjMYRI-2*)	TRINITY_DN9646_c1_g1_i1	OR167609.1	538	*Myrosinase ArMY1* (AY822710.1)	*Armoracia rusticana*	99%
				*TGG3* (OAO91400.1)	*Arabidopsis thaliana*	81%
				*Putative myrosinase 3* (XM_021024741.1)	*Arabidopsis lyrata*	78%
*Myrosinase isoform 3* (*WjMYRII*)	TRINITY_DN27880_c0_g1_i1	OR167610.1	516	*Myrosinase 4* (XM_006393050.2)	*Eutrema salsugineum*	89%
				*Myrosinase* (JN638573.2)	*Armoracia rusticana*	85%
				*Myrosinase 4* (XM_048750539.1)	*Brassica napus*	83%

Three *MYR* isogenes were identified, namely, *WjMYRI-1*, *WjMYRI-2*, and *WjMYRII*. As mentioned previously, the sequence of *WjMYRI-1* is identical to that of *MYR* cloned from wasabi petioles in a previous study (accession no. AB194903.1), whereas *WjMYRI-2* and *WjMYRII* (accession no. OR167609 and OR167610, respectively) are novel MYR-encoding genes identified in wasabi plant [17]. Predicted protein sequences showed high similarity to existing MYR protein sequences from other species.

In our investigation, transcriptomic data obtained from different plant organs of *W. japonica,* including leaves, roots, and seeds were included in the analysis [18]. Consequently, that would minimize the possibility of low expressed gene in specific organs as reported in *A. thaliana* [15].

Further analysis revealed that only six non-redundant hits were detected as shown in Supplementary Table S1 ~ S4. According to the BLASTN (*megablast*) results, augustus_masked-ctg477-processed-gene-1.6-mRNA-1 was the most homologous transcript to *WjMYRI* (both *WjMYRI-1* and *WjMYRI-2*) in *W. japonica* (Supplementary Table S1). However, there was no hit for *WjMYRII* gene sequence. The BLASTN (*blastn*) results suggested that maker-ctg375-augustus-gene-1.18-mRNA-1 was the best hit for *WjMYRII* showing less significant homology with a high E-value (Supplementary Table S2). Furthermore, the protein sequence of the best hit was not the best one from BLASTP results (Supplementary Table S3). The identical domain profile was predicted from the sequences of *WjMYRI-1* and *WjMYRII*, however, the protein sequences encoded by the six non-redundant transcript hits against *W. japonica* protein sequences did not show the identical profile (Supplementary Table S4). These observations revealed that augustus_masked-ctg477-processed-gene-1.6-mRNA-1 which was short in length (219 aa), might be incompletely predicted transcript for *WjMYRI*. On the other hand, no hits for *WjMYRII* were found in the transcript sequences of *W. japonica*.

By integrating BLAST results from transcriptome data of different organs and the transcript and protein sequences of *W. japonica* genome, it can be inferred that three myrosinase genes identified in this study (*WjMYRI-1*, *WjMYRI-2*, and *WjMYRII*) were mainly expressed for the myrosinase regulation and the probability of additional myrosinase genes is deemed very low.

### Genetic evolution of *Wasabia japonica*

Phylogenetic analysis was conducted using proteome data from *W. japonica* and other ten species in the Brassicaceae family to elucidate the genetic evolution of *W. japonica* within the cruciferous family. In line with previous findings, the results demonstrated a phylogenetically close relationship between *W. japonica* and other Brassicaceae species, particularly a high genetic similarity between *W. japonica* and *E. salsugineum* (Supplementary Fig. S1) [19, 20]. Moreover, the analysis indicated that *W. japonica* follows an independent evolutionary lineage from *B. juncea* and *A. rusticana* [18, 21]. Notably, although phylogenetic trees have been constructed using different data, including trnK/matK sequences, and chloroplast protein coding genes or CDS sequences in chloroplast genomes, we observed consistent results, underscoring the accuracy of our finding.

### Classification and evolutionary relationship of identified MYR isoforms

**Fig. 1:**
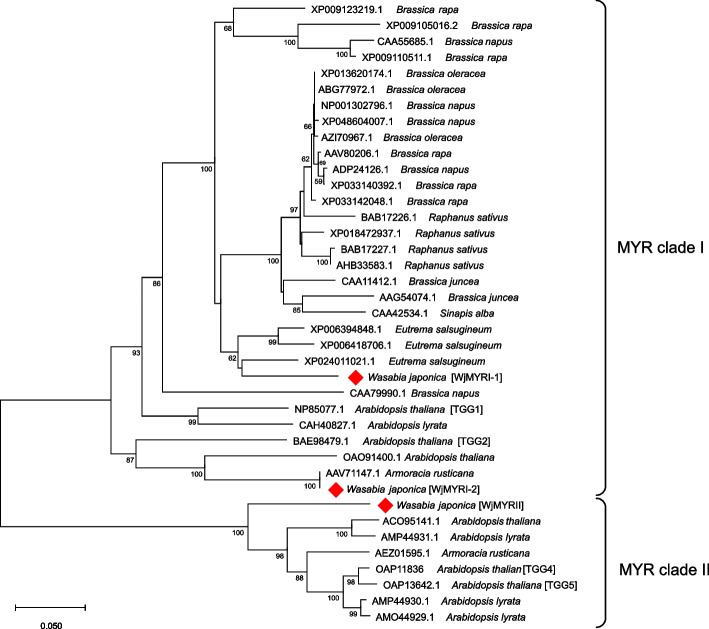
Consensus phylogenetic tree analysis revealing the relationship of identified myrosinase sequences with other myrosinase sequences found in Brassicaceae members. The analysis was conducted in MEGA ver. 11 with the neighbor-joining method. Bootstrap values less than 70% are not provided

Multiple sequence alignment using ClustalW revealed that the protein sequences of *WjMYRI-1* and *WjMYRI-2* exhibited high similarity to those of *AtTGG1* and *AtTGG2*, whereas the protein sequences of *WjMYRII*, *AtTGG4*, and *AtTGG5* displayed a greater abundance of highly conserved regions. The alignment scores of WjMYRII with TGG4 and TGG5 were 90%. As MYR belongs to the *ß*-glucosyl hydroxylase 1 family, the primary N-terminal signature was present in MYRs in both *W. japonica* and *A. thaliana* (Supplementary Fig. S2). Motifs such as “TINQP,” “TYITENG,” and “ITENG” were identified in TGG4, TGG5, and WjMYRII, whereas motifs such as “TINQL,” “IYVTENG,” “VTENG,” were observed in TGG1, TGG2, WjMYRI-1, and WjMYRI-2 (Supplementary Fig. S2). Phylogenetic tree analysis consistently revealed that WjMYRI-1 and WjMYRI-2 were closely related to TGG1 and TGG2 in *A. thaliana* and other MYRs clade I in other cruciferous plants, such as *B. juncea*, *B. oleracea*, *B. rapa*, and *S. alba* (Fig. 1). Similarly, WjMYRII showed close proximity to TGG4, TGG5, and other MYR sequences belonging to clade II. *AtTGG4* and *AtTGG5* were the first *MYR* genes to be identified in *A. thaliana*. *RsMyr2* and *ArMY2* have been characterized in the roots of radish and horseradish, respectively [22, 23].

### Expression of identified *MYR* isogenes in vegetative organs

**Fig. 2:**
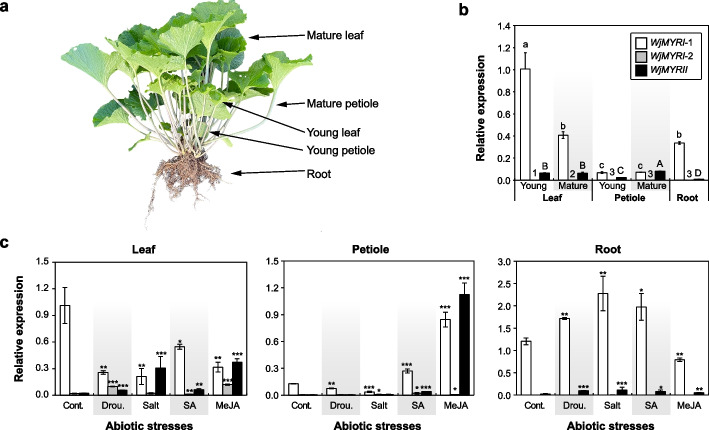
**a** Wasabi plant with roots, young and mature leaves, and petioles. **b** Quantitative real-time PCR analysis revealed the transcription levels of identified myrosinase isogenes in different organs of *Wasabia japonica* plant. Data were normalized to the value of *WjMYRI-1* in young leaves. Different superscripted numbers and letters indicate significant differences analyzed by one-way ANOVA, followed by Tukey’s test, *P* < 0.05. **c** Expression of identified myrosinase isogenes in different organs (leaves, petioles, and roots) of *W. japonica* plants subjected to abiotic stress. Data were normalized to the value of *WjMYRI-1* expressed in the leaves. Asterisk indicates significant difference between the treated and non-treated samples analyzed using two-tailed t-test, *P* < 0.05 (*), *P* < 0.01 (**), and *P* < 0.001 (***). “ns” denotes non-significant difference between the treated and non-treated groups analyzed with two-tailed t-test, *P* > 0.05. Abbreviations: Cont., control; Drou., drought; MeJA, methyl jasmonate; SA, salicylic acid

The transcript levels of *MYR* isogenes in different vegetative organs were investigated (Fig. 2a). Our results revealed that *WjMYRI-1* was the most abundantly expressed gene in wasabi plant. *WjMYRII* was predominantly found in the aboveground tissues, with lower expression than *WjMYRI-1*. Notably, *WjMYRI-2* was present at trace levels relative to *WjMYRI-1* and *WjMYRII* (Fig. 2b). Moreover*,* the expression of *WjMYRI-1* and *WjMYRII* significantly varied across various plant organs (*P* < *0.05*). Particularly, *WjMYRI-1* was highly expressed in the young leaves, but its expression was significantly lower in mature leaves and roots, and even approximately 10 times lower in young and mature petioles (*P* < *0.05*). Conversely, *WjMYRII* expression were significantly higher in leaves and mature petioles than young petioles and roots (*P* < 0.05).

In this study, mature leaves and roots exhibited similar levels of *WjMYRI-1*, whereas mature petioles possessed the lowest *WjMYRI-1* expression. The previous finding indicated that *WjMYRI-1* mRNA was comparably expressed in petioles and rhizomes, yet two times less in leaves [17]. Rather than technical errors, the discrepancy in organ-specific expression levels could result from various factors, such as plant growth conditions and stages. This is evident in the fluctuation of *RsMyr2* in different tissues during radish development [22]. In previous study, the production of rhizomes might lead to physiological changes in *W. japonica* in associated with gene regulation. In summary, those results suggested the flexible regulation of *MYR* genes in response to various factors.

### Regulation of *MYR* isogenes in response to abiotic factors

MYR plays a pivotal role in plant defense systems primarily because of its putative role in GSL degradation. Abiotic factors such as drought, salinity, and phytohormones were recognized for their influence on MYR activity as well as GSL production in previous findings. Particularly, salt concentrations (10 ~ 100 mM) were treated radish sprout and exhibited the inhibitory effect on MYR activity [24]. Besides, *T. salsuginea* was subjected to different sodium chloride concentrations (200 ~ 400 mM) to observe the impact of salinity on MYR-GSL system [25]. In previous study, the application of abscisic acid and MeJA at 100 µM significantly altered *MYR* gene expression in radish sprout [22]. Consequently, a solution of 300 mM sodium chloride was applied to induce salt stress in mature wasabi plant, while MeJA and SA at a concentration of 100 µM were utilized in phytohormone stimulation.

In wasabi, *MYR* genes respond differently to different stimuli and plant organs. In the leaves, *WjMYRI-1* expression was significantly downregulated in response to the abiotic treatments (*P* < 0.05). In particular, SA reduced *WjMYRI-1* expression by two-fold, whereas drought, salt, and MeJA lowered this expression by three-fold. In the petioles, *WjMYRI-1* expression was not affected by drought, downregulated by salt treatment, and upregulated by phytohormone application. In the roots, only MeJA treatment alleviated *WjMYRI-1* expression, whereas the other factors enhanced its expression, with salinity causing a two-fold upregulation (Fig. 2c). By contrast, *WjMYRI-2* expression showed no significant changes in the petioles and roots under abiotic stimuli, but it was significantly upregulated (*P* < 0.05) in the leaves under drought and MeJA treatments (Fig. 2c). Notably, *WjMYRII* was highly responsive to the stress-inducing factors. In the leaves, *WjMYRII* expression was upregulated by up to ten-fold under 300 mM NaCl treatment and by 16-fold under MeJA treatment. Drought and SA treatments doubled *WjMYRII* expression compared with that in the control samples (*P* < 0.05). Notably, *WjMYRI-1* expression was downregulated in the treated leaf tissues, whereas *WjMYRII* expression in leaves was upregulated by abiotic treatments. Among the abiotic stresses applied, MeJA significantly induced the expression of *WjMYRII* in the leaves and petioles.

### GSL production in *W. japonica*

**Fig. 3:**
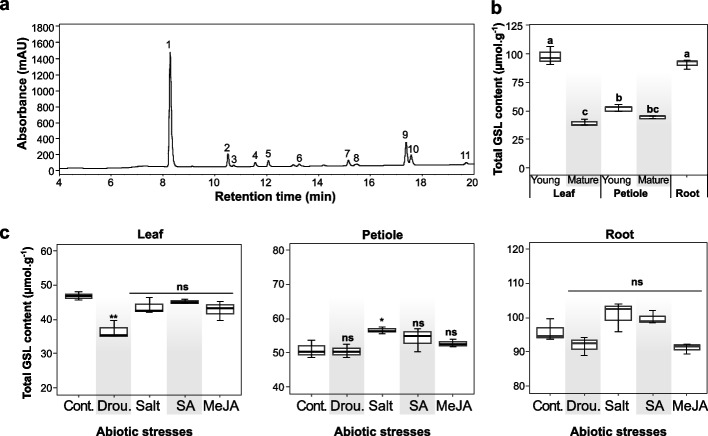
**a** Glucosinolates identified in *Wasabia japonica* extract by using high-performance liquid chromatography-electrospray ionization tandem mass spectrometry (HPLC-ESI/MS). **1**- sinigrin, **2**- glucoalyssin, **3**- glucohesperin, **4**- glucoibervirin, **5**- 7-(methylsulfinyl)heptyl glucosinolate, **6**- glucotropaeolin (internal standard), **7**- methoxyglucobrassicin, **8**- glucoraphanin, **9**- neoglucobrassicin, **10**- glucolesquerellin, and **11**- glucoarabishirsutain. **b** Total glucosinolate content (µmol‧g^−1^) in different vegetative organs harvested at 56 days after planting. **c** Total glucosinolate contents (µmol‧g^−1^) in the leaves, petioles, and roots exposed to abiotic stimuli. Different letters indicate significant difference among plant organs analyzed with one-way ANOVA, followed by Tukey’s test, *P* < 0.05. “ns” denotes non-significant difference between the treated and non-treated samples analyzed with two-tailed t-test, *P* > 0.05. Abbreviations: Cont., control; Drou., drought; GSL, glucosinolate; MeJA, methyl jasmonate; SA, salicylic acid

In this study, HPLC-ESI/MS results revealed the enrichment of GSL in the wasabi extract (Fig. 3a). Ten individual GSLs were identified, including sinigrin, glucohesperin, glucoraphasatin, glucoibervirin, 7-(methylsulfinyl)heptyl GSL, methoxyglucobrassicin, glucoraphanin, neoglucobrassicin, glucolesquerellin, and glucoarabishirsutain, which are commonly found in wasabi [26]. Aliphatic GSLs accounted for over 90% of the total GSL content in the wasabi samples (Supplementary Table S5). Furthermore, the total GSL content varied among the different plant organs of wasabi (Fig. 3b). Particularly, the highest total GSL content was obtained from the young leaves and roots (97.89 and 91.17 µmol‧g^−1^, respectively), whereas the young petioles contained approximately 54.14 µmol‧g^−1^. Additionally, GSL accumulation was influenced by tissue maturity, with the mature leaves and petioles producing lower amounts of GSL content (38.36 and 44.79 µmol‧g^−1^, respectively) than their younger counterparts. These findings align with those of previous studies that reported variations in GSL accumulation across various plant organs. For example, Pang et al. [25] reported that the GSL production is varied across roots, leaves, flowers, and siliques of *Thellungiella salsuginea*. Along with the transition of growth stages, younger leaves of *A. thaliana* contained higher GSL concentration than older leaves [27]. Furthermore, previous research indicated that indolic GSLs prominently accumulate in the belowground parts [28]. In wasabi plants, indolic GSL content was approximately 10–20 times higher in the roots than in the mature petioles and leaves (Supplementary Table S5). In most cruciferous plants, such as broccoli, cabbage, and cauliflower, the contents of aliphatic GSLs often exceed those of indolic GSLs [29].

The compositions of ten major GSLs are shown in Supplementary Table S5. Notably, sinigrin was consistently the most prevalent GSL, constituting greater than 80% of the total GSL content. Glucoibervirin, glucohesperin, and glucosequerellin were also present in considerable quantities in the wasabi extract; however, their contents were much lower than that of sinigrin. Previous studies have consistently identified sinigrin as the most abundant GSL in various wasabi plant parts, including the leaves, flowers, seeds, roots, and rhizomes [26, 30–32]. Our findings are aligned with previous study, indicating a higher abundance and accumulation of sinigrin in the rhizomes and roots than in the leaves and petioles [32].

### Effects of abiotic factors on glucosinolate production in wasabi plant

The abiotic treatments did not show the impact on GSL composition in the leaves, petioles, and roots (Supplementary Table S6). Total GSL content was not significantly affected by most of abiotic treatments (*P* > 0.05, Fig. 3c). Only drought significantly decreased the total GSL content in the leaves, whereas salt treatment significantly increased the total GSL content in the petioles attributed to the increase of glucoibervirin and sinigrin (*P* < 0.05).

Despite the non-significant change in the total GSL content, we observed significant changes in certain GSL compounds (*P* < 0.05). Particularly, sinigrin content in the leaves was reduced, whereas glucohesperin production in the petioles and roots was adversely affected by abiotic treatments. Drought reduced glucoibervirin and glucolesquerellin levels, but the other factors significantly enhanced their accumulation (*P* < 0.05).

Previous findings indicated that GSL production in other Brassicaceae species is markedly influenced by biotic and abiotic stresses. For example, drought for two weeks increases the total content of GSLs in broccoli and promotes the accumulation of individual GSLs, such as neoglucobrassicin, glucobrassicin, and progoitrin [33]. Similarly, water deficiency also significantly varies GSL accumulation in Chinese cabbage and pak choi [34, 35]. Exogenous phytohormone can affect GSL content in Chinese cabbage [36]. Treatment with 40 and 80 mM sodium chloride significantly increases the total GSL content in broccoli after 1 and 15 days [37]. However, limited research has been conducted on the environmental effects on GSL biosynthesis in wasabi thus far.

### Identification and quantification of GHPs

**Fig. 4:**
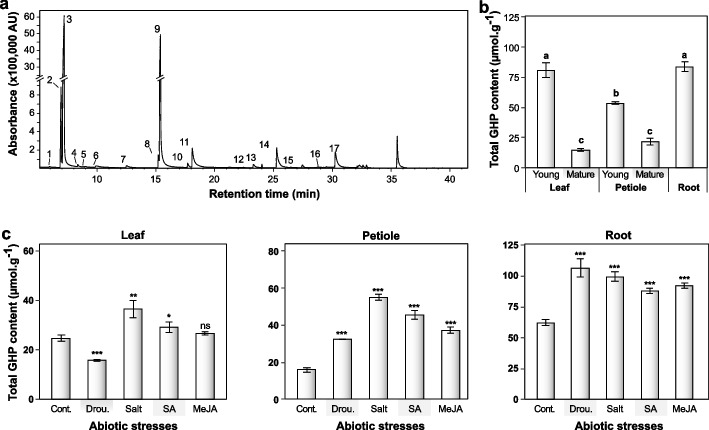
**a** Glucosinolate hydrolysis products identified in *Wasabia japonica* roots by using gas chromatography-mass spectrometry (GC–MS). **1**- Isopropyl isothiocyanate, **2**- allyl thiocyanate, **3**- Allyl isothiocyanate, **4**- isobutyl isothiocyanate, **5**- Butyl isothiocyanate, **6**- 3-butenyl isothiocyanate, **7**- 4-pentenyl isothiocyanate, **8**- 5-hexenyl isothiocyanate, **9**- phenyl isothiocyanate (internal standard), **10**- heptenyl isothiocyanate, **11**- ibervirin, **12**- beteroin, **13**-unknown, **14**- lesquerellin, **15**- 1-(methyl)indole-2-carboxylic acid, **16**- 7-(methylthio)heptyl isothiocyanate, and **17**- hesperin. **b** Total hydrolysis product content (µmol‧g^−1^) in different vegetative organs harvested at 56 days after planting. **c** Variation in total hydrolysis product content (µmol‧g^−1^) in the leaves, petioles, and roots exposed to abiotic stimuli. Different letters present the significant difference among plant organs analyzed with one-way ANOVA, followed by Tukey’s test, *P* < 0.05. Asterisks display significant difference between the treated and control samples analyzed with two-tailed t-test, *P* < 0.05 (*), *P* < 0.01 (**), *P* < 0.001 (***). “ns” denotes non-significant difference between the treated and non-treated groups analyzed with two-tailed t-test, *P* > 0.05. Abbreviations: Cont., control; Drou., drought; GHP, glucosinolate hydrolysis product; MeJA, methyl jasmonate; SA, salicylic acid

As mentioned earlier, MYRs are responsible for the primary conversion of GSLs into different GHPs, which form a system that protects host plants against stress, herbivores, phytopathogens, and environmental influences [38, 39]. Hence, we sought to investigate the formation of GHPs to elucidate the distinct regulation of MYR clades I and II in *W. japonica*. In the current study, the following 15 hydrolysis compounds were identified in the wasabi extract using GC–MS/MS: isopropyl isothiocyanate, allyl thiocyanate, allyl isothiocyanate, isobutyl isothiocyanate, butyl isothiocyanate, 3-butenyl isothiocyanate, 4-pentenyl isothiocyanate, 5-hexenyl isothiocyanate, heptenyl isothiocyanate, ibervirin, beteroin, lesquerellin, 1-(methyl)indole-2-carboxylic acid, 7-methylthioheptyl isothiocyanate, and hesperin (Fig. 4a). Isothiocyanates were abundant in all the samples; particularly, allyl isothiocyanate, which serves as a principal product of sinigrin conversion. Other GHPs were present in low or trace amounts, especially in the mature leaves (Supplementary Tables S7 and S8). We did not observe some corresponding GHPs of the detected GSLs in the wasabi extract. In agreement with previous study, many minor isothiocyanates were enriched, but their precursors were not present at detectable range [26]. The mismatch between the GSL and GHP profiles may be ascribed to differences in the extraction process and analytical instrument sensitivity.

As shown in Fig. 4b–c, the total relative GHP content produced from endogenous GSLs in different wasabi samples was determined. Consistent with the GSL quantification results, the young leaves and roots produced the highest GHP concentration (approximately 80 µmol‧g^−1^), followed by the young petioles (approximately 53 µmol‧g^−1^). The mature leaves and petioles released less than 25 µmol‧g^−1^ GHP (Fig. 4b). Supplementary Table S7 shows the composition and content of individual GHPs identified in the different plant tissues. Total GHP content was mainly contributed by allyl thiocyanate, allyl isothiocyanate, lesquerellin, hesperin, and ibervirin. Most GHPs were detectable in the roots, whereas many compounds were not detected in the mature leaves and petioles (Supplementary Table S7). The young leaves yielded approximately 72.07 ± 7.51 µmol‧g^−1^ allyl isothiocyanate, whereas the roots contained approximately 69.31 ± 4.89 µmol‧g^−1^ allyl isothiocyanate.

Despite causing minor changes in total GSL content, the abiotic treatments significantly affected the total GHP content (*P* < 0.05). Particularly, salinity resulted in nearly double and triple GHP concentrations in the leaves and petioles, respectively, compared to their control counterparts. In addition, the treated roots produced higher GHP concentrations (~ 100 µmol‧g^−1^) than the control roots (~ 60 µmol‧g^−1^). The significant change in total GHP content was mostly attributed to the effect of the abiotic treatments on the production of allyl isothiocyanate (Supplementary Table S8). The elevated concentrations of GHPs in the stress-induced samples suggest its potential roles in the plant defense system. Unfortunately, the effects of abiotic factors on GHP production have not been studied as extensively as their precursors. Examination of GSL accumulation and GHP formation indicated that subjecting wasabi plants to abiotic stresses before harvesting would alter their flavor perception and pharmaceutical value.

## Discussion

### Identification of different *MYR* isogenes in *W. japonica*

Three potential isogenes (*WjMYRI-1*, *WjMYRI-2*, and *WjMYRII*) encoding for different MYR isoforms in wasabi were identified. *WjMYRI-2* and *WjMYRII* are novel *MYR* genes identified in *W. japonica*. The outcomes of sequence alignment and phylogenetic tree analyses indicated that *W. japonica* possessed regular MYRs in clades I and II (Fig. 1). So far, limited studies focused on the identification, characterization, and function of MYRs in clade II. Consequently, it is important to simultaneously investigate MYR clades I and II in wasabi. In clade I, *WjMYRI-1* was identified as the principal gene present in all plant organs, whereas *WjMYRI-2* showed only trace expression levels. This result suggested that *WjMYRI-2* may be a pseudogene or non-functional gene. In clade II, *WjMYRII* (clade II) was highly expressed in aboveground vegetative tissues.

In *A. thaliana*, MYRs in clade I are widely present across various plant organs, whereas MYRs in clade II are mostly found in the underground roots. *AtTGG1* and *AtTGG2* are exclusively detected in the leaves and stems, whereas *AtTGG4* and *AtTGG5* are solely expressed in the roots [15]. In the present study, *WjMYRII* showed opposite expression patterns despite the high sequence identity with TGG4 and TGG5 at amino acid level (Supplementary Fig. S2). In radish, *RsMyr1* (clade I) is also present in all plant organs, whereas *RsMyr2* (clade II) was first isolated and characterized from the roots of radish plant [22]. However, the expression of *RsMyr2* is varied across plant organs and growth stages. From the cotyledon stage to the taproot thickening stage, *RsMyr2* is highly expressed in the leaves, followed by the stems and roots. As the taproot reached maturity, *RsMyr2* transitions from the leaves to the stems and roots.

Although MYR clade I is predominantly found in the Brassicaceae family, MYR clade II is believed to be an ancestral form of MYR, which is widely present in the plant kingdom. For example, in *Carica papaya*, at least three *MYR* isogenes classified in clade II possess distinct expression patterns. Specifically, *CpTGG1* is detected in the aboveground tissues, *CpTGG2* is solely expressed in the roots, and *CpTGG3* expression is restricted in the seeds and flowers [40]. These findings revealed that although various MYRs are concurrently found in a species, their expression varies across plant organs and growth stages. Furthermore, MYRs in clades I and II may undergo different transcriptional regulation and be responsible for specific regulatory functions.

Consistently, abiotic treatments significantly altered *MYR* gene expression across various wasabi plant organs. Notably, *WjMYRII* expression was predominantly upregulated in response to abiotic stimuli. Unfortunately, the regulatory functions of MYRs in response to stress-inducing factors remain obscured. Previous studies have primarily focused on investigating the effect of abiotic factors on MYR activity [25]. For instance, salt treatment at four concentrations causes random alterations in MYR activity in the leaves, flowers, and siliques of *T. salsuginea*. Another study demonstrated the inhibitory effects of salt stress on MYR activity in radish sprouts. These findings highlight the complex response of MYRs to saline treatment, which may vary across different organs and growth stages. The following research outcomes demonstrate the impact of abiotic stimuli on *MYR* genes of Brassicaceae plants. Cocetta et al. [41] reported that high salinity and heat treatment do not affect the expression of the MYR-encoding gene *Dtmyro* in *Diplotaxis teneuifolia* L. Yan et al. [22] indicated that *RsMyr2* expression is upregulated in the leaves of radish plants subjected to different stress-inducing factors, including wounding, abscisic acid, MeJA, and peroxide treatments. While wounding downregulates *RsMyr2* expression in the leaves, other stimuli gradually upregulates *RsMyr2* expression.

### Putative roles of identified MYRs in GSL metabolism

**Fig. 5:**
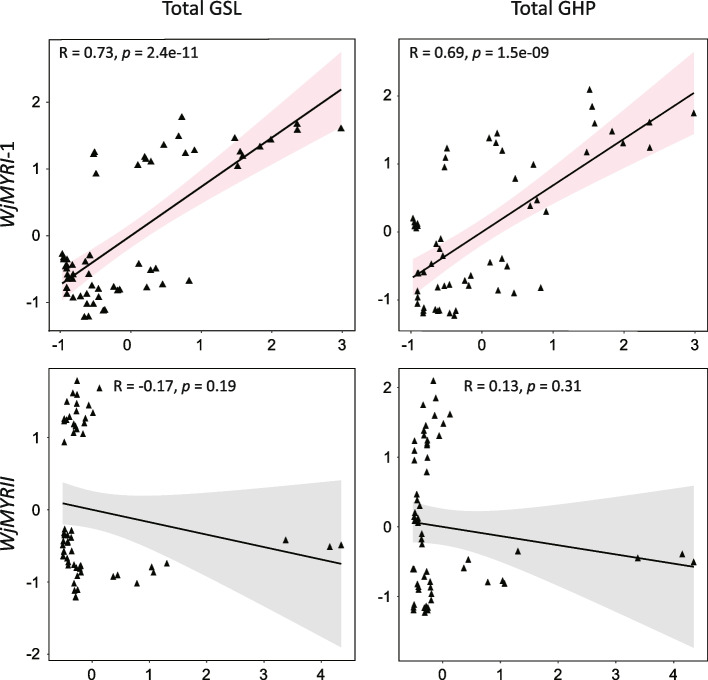
Correlation between total glucosinolate content, hydrolysis product content, and relative expression of *WjMYRI-1* and *WjMYRII*. Correlation efficiency (R) was determined with Pearson correlation analysis

MYRs and GSLs are independently synthesized and compartmentalized in different tissues. However, their coexistence in nature implies a common biological evolution. We observed a positive correlation between GSL accumulation and *WjMYRI-1* expression in different plant organs (Fig. 5). Particularly, *WjMYRI-1* was highly expressed in the GSL-enriched young leaves and roots (Figs. 2b and 3b). In *S. alba*, leaves have higher GSL concentration and MYR activity than stems possibly because of their greater susceptibility to pest attacks [42]. RsMYR2 (clade II) in young radish roots may have a strong affinity for gluconasturtiin [23], suggesting MYR expression can be correlated with the preference of the GSL substrate. Given the overlapping GSL profiles of wasabi leaves, petioles, and roots, exploring the substrate preferences of WjMYRI-1, WjMYRI-2, and WjMYRII may be challenging. Further studies on the specific catalytic characteristics of MYR isoforms in *W. japonica* are required.

The MYR–GSL system is primarily responsible for plant defense against the impact of environmental factors. In the present study, we determined the regulatory functions of MYRs in clades I and II via the activity of the MYR–GSL system in response to the abiotic treatments. Although the abiotic treatments considerably altered the *MYR* expression levels, they did not significantly affect GSL production. Under stress conditions, *WjMYRI-2* showed independent regulation, whereas *WjMYRI-1* and *WjMYRII* showed similar patterns in the petioles and roots but at different levels. Most of the abiotic treatments significantly upregulated *WjMYRII* expression. Reduction in MYR activity is essential for GSL retention in tissues under stress conditions. In the present study, *WjMYRI-1* expression significantly reduced only in the leaves. This finding might align with the theory of optimal defense as leaf tissue is more vulnerable to stress.

**Fig. 6:**
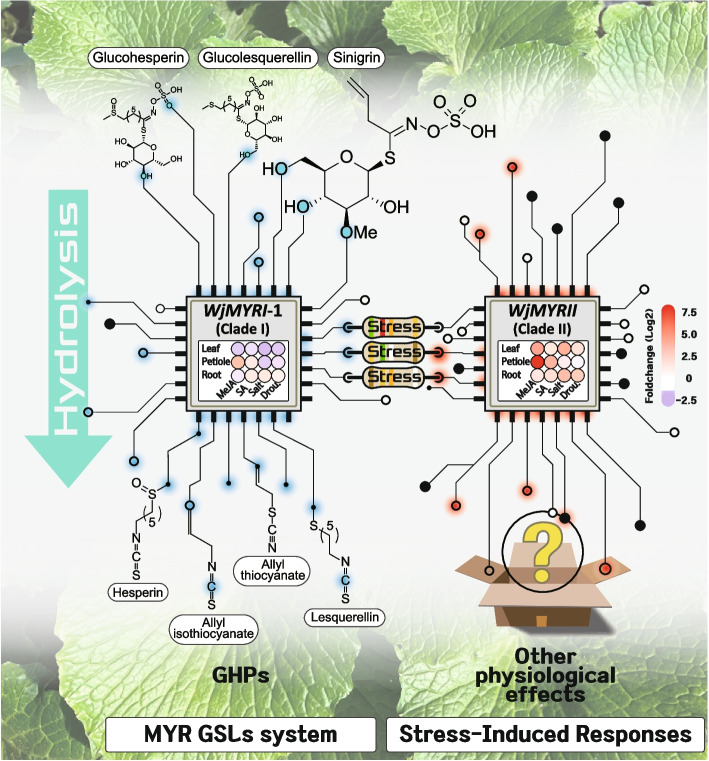
Theoretical roles of MYR isoforms in glucosinolate metabolism and stress-induced responses. Abbreviations: GHP, glucosinolate hydrolysis product; GSL, glucosinolate

GSLs and GHPs showed the strongest correlation (R ~ 0.9, Supplementary Table S9). Notably, the total GHP content in most samples was significantly higher than that in the control samples (*P* < 0.05). Given that GHP formation may be controlled by other proteins and cofactors, confirming whether the increased GHP levels resulted from MYR activity in response to stress may be difficult. Further studies are required to specifically examine the physiological characteristics of distinct MYRs. Nevertheless, we observed a positive correlation between GHP formation and *WjMYRI-1* expression, suggesting a key role for WjMYRI-1 in GSL degradation in *W. japonica* (Fig. 5). The expression of *WjMYRII* was not correlated with either GSL production or GHP formation but was highly regulated by the abiotic treatments (Fig. 6). In *A. thaliana*, MYRs in clades I and II have evolved to possess different functions and are involved in plant physiological pathways other than GSL conversion. In particular, TGG1 and TGG2 may redundantly regulate ABA and MeJA signaling to induce stomatal closure, which is critical for constraining the impact of water shortage. TGG1 and TGG2 (clade I) may share redundant functions in the conversion of GSL upon the damage caused by insects [38]. Double mutant *tgg1tgg2* of *A. thaliana* impairs GSL hydrolysis. In *A. thaliana*, the mutant *tgg4tgg5* fails to elongate the root system under natural conditions, indicating the critical role of TGG4 and TGG5 (Clade II) in auxin biosynthesis, which regulates root growth [43, 44].

## Conclusions

In conclusions, three potential isogenes (*WjMYRI-1*, *WjMYRI-2*, and *WjMYRII*) encoding for different MYR isoforms in *W. japonica* were identified. *WjMYRI-2* and *WjMYRII* are novel *MYR* genes identified in *W. japonica*. Furthermore, the *MYR* isogenes were further classified into clades I and II based on the results of multiple sequence alignment and phylogenetic analyses. The present study is the first to report MYR clade II in *W. japonica*. Our results shed light on the difference between MYRs in clades I and II. Particularly, *WjMYRI-1* may be primarily responsible for GSL degradation, whereas *WjMYRII* (clade II) may be involved in other regulatory pathways induced by abiotic factors. In the future, further studies are still warranted to characterize their functions in GSL degradation or other stress-induced pathways by employing bioengineering approaches such as gene overexpression and gene silence.

## Materials and methods

### Chemicals

Absolute ethanol, allyl isothiocyanate, barium acetate, *ß*-mercaptoethanol, cetyltrimethylammonium bromide (CTAB), chloroform, ethylenediaminetetraacetic acid (EDTA 500 mM), formic acid (FA), isoamyl alcohol, lead(II) acetate, phenyl isothiocyanate, polyvinylpyrrolidone (PVP), sodium acetate, sodium chloride, and Tris–HCl pH 7.5 were purchased from Sigma-Aldrich (St. Louis, MO, USA). Aryl sulfatase from *Helix pomatia* with 10,000 units (type H-1; EC 3.1.6.1) was obtained from Sigma-Aldrich. DEAE Sephadex A-25 was purchased from Cytiva (Marlborough, MA, USA). Glucotropaeolin potassium salt was obtained from Extrasynthese (Genay Cedex, Rhône, France). Plant Total RNA Mini Kit without DNase was purchased from Geneaid (New Taipei City, Taiwan). High-performance liquid chromatography (HPLC) solvents acetonitrile and methanol were purchased from Daejung (Republic of Korea), and water and dichloromethane were obtained from Thermo Fisher Scientific (Waltham, MA, USA).

### De novo assembly and annotation

In a previous study, the transcriptomes of wasabi leaves, stems, and roots were analyzed, and data have been deposited in Sequence Read Archive (https://www.ncbi.nlm.nih.gov/sra/) under the Bioproject PRJNA67067 with accession numbers SRX9404179, SRX9404181, and SRX9404180, respectively [18]. *MYR* isogenes were identified following the standard protocol available at Seeders, Republic of Korea (http://www.seeders.co.kr/). Briefly, raw sequencing reads were downloaded and pre-processed using the Trimmomatic software. The processed clean reads were assembled using Trinity, a powerful tool used for reconstructing the transcriptome without a robust model genome [45]. Transcriptome annotation was carried out using Trinotate Toolkit v2.01. BLASTX filter was used to search for functional protein-coding regions against MYR sequences (TGG) characterized in *A. thaliana* with E-value ≤ 1e-50 and identity > 70%.

Furthermore, to validate the de novo assembly results, we performed additional analysis using transcript and protein sequences assembled from *W. japonica* genome provided in the previous study [46]. Briefly, nucleotide and amino acid sequences of three *MYR* genes were searched against the transcript and protein sequences of *W. japonica* by using BLASTN and BLASTP, respectively [47]. InterProScan [48] was utilized to predict protein domains from the amino acid sequences of BLASTP hits (accessed on November 3, 2023).

### Phylogenetic analysis

Proteome data were retrieved from the NCBI GenBank/RefSeq database, excluding three specified species. Proteomes for *A. rusticana* and *W. japonica* were acquired from a public repository, as indicated in the respective data availability statements from the publications [46, 49]. The proteome file for *B. juncea* was obtained from Ensembl Plants database [50]. The proteome of *Chlorella variabilis* NC64A was included as an outgroup. A phylogenomic tree was generated by using CVTree, incorporating whole proteome sequences and employing a *K*-tuple length of seven [51, 52].

### Evolutionary relationship and classification of identified myrosinases

Predicted protein sequences of the identified *MYR* isogenes were obtained using Open Reading Frame Finder and basic local alignment search tool (BLASTX available at the National Center for Biotechnology Information (NCBI) https://www.ncbi.nlm.nih.gov/) to search for open reading frames and verify functional protein sequences, respectively. The predicted protein sequences of *WjMYRI-1*, *WjMYRI-2*, and *WjMYRII* were aligned with those of TGG1, TGG2, TGG4, and TGG5 obtained from the NCBI database and TAIR (http://www.arabidopsis.org/). Multiple sequence alignments were performed using ClustalW (https://www.genome.jp/tools-bin/clustalw).

The evolutionary relationship between MYRs identified in *W. japonica* and MYR sequences reported in other Brassicaceae plants (including *A. thaliana*) was unraveled by using molecular evolutionary genetics analysis (MEGA) software version 11 (http://megasoftware.net). The putative MYR protein sequences from other Brassicaceae species were collected from either NCBI/protein database or the Brassica database. After a condensed alignment using MEGA version 11, a phylogenetic tree was constructed using the neighbor-joining method with 1000 bootstrap replicates and complete deletion mode.

### *Wasabia japonica* cultivation and treatment of abiotic factors

Wasabi plants were grown under greenhouse conditions at the Agricultural Research & Extension Services, Gangwon-do, Republic of Korea. At 56 days after transplanting, five wasabi plants were harvested and subdivided into young and mature leaves, petioles, and roots to investigate MYR expression in different vegetative organs.

At 63 days after transplanting, the plants were subjected to different abiotic stresses, encompassing drought, salinity, SA, and MeJA. Briefly, water was withheld for a period of two weeks to simulate drought. A solution of 300 mM sodium chloride was applied at 24 h before sampling to trigger salt stress. Phytohormones in the form of 100 µM SA and MeJA solutions were sprayed 6 h prior sampling. Five biological replicates were collected and combined for further analyses. Each wasabi plant was divided into mature leaves, petioles, and roots. All samples were flash frozen in liquid nitrogen and stored at -80 °C. Freeze-dried powder samples were used for GSL and GHP analyses.

### Isolation of RNA from wasabi samples

Samples were finely ground with liquid nitrogen in mortars and stored at -80 °C. RNA was isolated from wasabi samples by using two protocols for aboveground and underground samples. Particularly, root samples were extracted with the RNeasy Mini kit (Qiagen, Chatsworth, CA, USA) using RLC buffer, which is specially used for the RNA purification of carbohydrate-rich samples. RNA was isolated and purified in accordance with the manufacturer’s instructions with minor modifications. Compared with the roots, the leaves and petioles contained higher polyphenol contents, which contributed to the poor quality of RNA isolated using commercial kits. Therefore, RNA was isolated from other aboveground samples and then purified using the CTAB method in conjunction with the Geneaid kit. The CTAB buffer was prepared by mixing 2% (w/v) CTAB, 1.4 M sodium chloride, 20 mM EDTA, and 100 mM Tris–HCl at pH 7.5. Before extraction, CTAB buffer was added with 2.5% (w/v) PVP and then heated at 65 °C. Finally, 2.5% (v/v) *β*-mercaptoethanol was added to the hot mixture to prepare the isolation buffer. Next, 1 mL of isolation buffer was added to each sample tube and heated at 65 °C for approximately 12 min. The samples were centrifuged at 14,000 × g for 5 min at 4 °C to collect all supernatants. An equal volume of a mixture of chloroform/isoamyl alcohol (24/1, v/v) was added and vortexed vigorously. The aqueous layer was separated by centrifugation at 14,000 × g for 15 min at 4 °C. Half the volume of cold ethanol was added and mixed immediately with a micropipette. RNA was collected and purified using the GeneAid kit in accordance with the manufacturer’s instructions. Total RNA concentration was measured using a NanoDrop ND-1000 (Nano Drop Technologies, Thermo Fisher Scientific).

### Quantitative real-time polymerase chain reaction (qRT-PCR)

Total RNA (1 µg) was used to synthesize first strand cDNA using TOYOBO ReverTra Ace–α (TOYOBO, Osaka, Japan). The transcript expression levels of each *MYR* isogene in different organs, growth stages, and in response to different abiotic effects were determined using qRT-PCR. Primer sets were designed and generated by Bioneer (https://www.bioneer.co.kr/; Daejeon, Republic of Korea). Details of the primers used are provided in Supplementary Table S10. Each PCR contained LightCycler® 480 SYBR Green I Master Mix (Roche Diagnostics, Mannhein, Germany), PCR-grade water, and cDNA. The temperature program was set as follows: (1) preincubation (95 ℃ for 5 min), (2) cycling (95 ℃ for 10 s, 60 ℃ for 30 s), and (3) melting curve (95 ℃ for 10 s, 65 ℃ for 1 min, 97 ℃ for 1 s). *WjTUB-β6* was used as the reference gene for relative quantification. The Livak method (2^−ΔΔC^_T_) was used to calculate the relative transcript levels of *MYR* isogenes [53]. Three technological replicates were used for each sample. Samples from different organs were normalized to *WjMYRI-1* in the young leaves, whereas samples from different abiotic stress treatments were normalized to *WjMYRI-1* in the control leaves.

### Identification and quantification of glucosinolates

Desulfo-GSL (dsGSL) formation followed by HPLC-diode array detection (DAD) analysis was employed to determine GSL content. First, GSLs were extracted from 50 mg of freeze-dried wasabi samples using 2 mL of 70% (v/v) methanol. The samples were vortexed, heated at 90 ℃ for 20 min, and then sonicated at 70 ℃ for 15 min. The supernatant was collected via centrifugation at 3000 × g for 15 min at 4 ℃. Meanwhile, a mixture of 15 μL 1 mM glucotropaeolin as the internal standard (IS), 75 μL of 1 M barium acetate, and 75 μL of 1 M lead (II) acetate was prepared in a 2 mL microtube. After centrifugation, 1.2 mL of the supernatant was added to the mixture and then vortexed thoroughly before centrifugation at 12,000 × g for 10 min. The supernatant was loaded onto a Sephadex A25 column. Sulfatase H1 (Sigma-Aldrich, St. Louis, MO, USA) was used to cleave the sulfate groups. After adding 200 μL of purified sulfatase diluted in water, the column was left overnight for desulfation. Next, dsGSLs were eluted using HPLC-grade water, filtered into analytical vials, and then identified by adopting the method described by [54]. Briefly, an HPLC–DAD 1200 series system coupled with a quadrupole liquid chromatography/mass spectrometry 6120 model (Agilent Technologies, Santa Clara, CA, USA) was used. A 20 µL of extract samples and standard was injected at a flow rate of 0.7 µL‧min^−1^ into ODS-AQ C_18_ column (ID × L, 4.6 mm × 150 mm, 5 µm, YMC, Kyoto, Japan). Column temperature was controlled at 35 ℃, and the DAD signal was collected at a wavelength of 229 nm. The mobile phase included solvent A (0.1% FA added water) and solvent B (0.1% FA added acetonitrile). The analysis started at 98% solvent A for 2 min, increased to 65% within 14 min, decreased to 35% within 4 min, and continuously decreased to 0% at 22 min. The column was then washed with 100% solvent B for 8 min. The total running time was 30 min, and the post-running time was 5 min. DsGSLs were identified using positive electrospray ionization tandem mass spectrometry (ESI–MS) with a scanning mode ranging from 100 m*/z* to 1500 m*/z* to collect all mass spectral fragments. Individual GSLs present in the wasabi extract were identified by comparison with reference mass spectral data reported by [55]. GSL content was quantified using a standard curve of desulfo-glucotropaeolin constructed with corresponding peak areas at three concentrations (12.5, 25, and 50 µg‧mL^−1^). The quantification method has been described in detail in a previous study [54]. Three technological replicates were used for each sample.

### Identification and quantification of glucosinolate hydrolysis products

GHPs were identified and quantified using gas chromatography (GC) Agilent model 7980A coupled with a 5975C detection system (Agilent Technology, Inc., Santa Clara, USA). Approximately 150 mg of the freeze-dried samples was added with 3 mL of water and then incubated for 1 h at 37 °C to ensure a complete hydrolysis reaction between GSLs and endogenous MYRs in wasabi. GHPs were extracted by adding 0.6 mL of dichloromethane and 10 mM phenyl isothiocyanate (IS). After centrifugation at 3000 × g for 5 min, the upper aqueous layer was discarded, and the lower organic layer was collected. The DCM extract was filtered into an analytical vial and then subjected to GC–MS following a previously described method with minor modifications [26]. Samples and standards (2 μL) were injected into an HP-5MS column (30 m × 250 µm × 0.25 µm). The inlet and column line temperatures were set to 250 °C and 275 °C, respectively. The initial temperature was set to 40 °C, held for 2 min, and then increased by 270 °C at a rate of 6 °C‧min^−1^. The post-running time was set to 5 min at 40 °C. Helium was flowed at 2 mL‧min^−1^. The scan mode ranged from 30 to 300 m/z at 70 eV, 5.1 scans/sec. GHPs were identified using the mass spectral databases available at the National Institute of Standards and Technology (https://www.nist.gov/) and PubChem (https://pubchem.ncbi.nlm.nih.gov/). Allyl isothiocyanate content was calculated using a standard curve constructed with an allyl isothiocyanate standard at concentrations of 5, 10, and 20 mM. The contents of the other identified GHPs were measured using a standard curve constructed with a phenyl isothiocyanate standard at concentrations of 3.5, 7, and 14 mM. The relative GHP concentration was determined using the following formula:

$$C_{GHP}=\frac{A_{GHP}}{A_{IS}}\times C_{IS}(\mathrm\mu\mathrm g\cdot\text{mL}^{-1})$$

Where *A* is the peak area, and *C* is the concentration.

### Correlation and statistical analyses

After data standardization, Pearson correlation analysis was performed using the function available in Microsoft Excel and visualized with the ggplot2 and ggpubr packages available in R version 4.1.2. All data are presented as mean ± standard deviation. Statistical analyses were performed using GraphPad Prism version 7.03. One-way ANOVA was performed, followed by Tukey’s test, *P* < 0.05. A two-tailed t-test was performed between the treatment and control groups with a 95% confidence interval, *P* < 0.05.

### Supplementary Information

**Supplementary Material 1.**

## Data Availability

All data generated or analysed during this study are included in this published article and its supplementary information files.

## Abbreviations

GSL: Glucosinolate
MYR: Myrosinases
SSR: Short sequence reading
GHP: GSL hydrolysis product
CTAB: Cetyltrimethylammonium bromide
EDTA: Ethylenediaminetetraacetic acid
FA: Formic acid
PVP: Polyvinylpyrrolidone
HPLC: High performance liquid chromatography
BLASTX: Basic local alignment search tool
NCBI: National Center for Biotechnology Information
MEGA: Molecular evolutionary genetics analysis
SA: Salicylic acid
MeJA: Methyl jasmonate
qRT-PCR: Quantitative real-time polymerase chain reaction
dsGSL: Desulfo-GSL
DAD: Diode array detection
ESI–MS: Electrospray ionization tandem mass spectrometry
GC: Gas chromatography
IS: Internal standard
