# Supplementary material for: Myrosinase isogenes in wasabi (Wasabia japonica Matsum) and their putative roles in glucosinolate metabolism
Source: BMC Plant Biol. 2024 May 1;24:353. doi: 10.1186/s12870-024-05057-w (PMC11061951; doi:10.1186/s12870-024-05057-w)
Supplement: Supplementary file 1 — Supplementary Material 1. [file 12870_2024_5057_MOESM1_ESM.docx]

**Myrosinase isogenes in wasabi (*Wasabia* *japonica* Matsum) and their putative roles in glucosinolate metabolism**

To Quyen Truong^1,2^, Yun Ji Park^2^, Je-Seung Jeon^3^, Jaeyoung Choi^4^, Song Yi Koo^5^, Yeong Bin Choi^2^, Phuong Kim Huynh^1,2^, Jinyoung Moon^2^, Sang Min Kim^1,2*^

^1^ Division of Bio-Medical Science & Technology, Korea Institute of Science and Technology (KIST), University of Science and Technology, Seoul 02792, Republic of Korea

^2^ Smart Farm Research Center, KIST Gangneung Institute of Natural Products, Gangneung 25451, Republic of Korea

^3^ Department of Herbal Crop Research, National Institute of Horticultural and Herbal Science, RDA, Eumseong 27709, Republic of Korea

^4^ Department of Oriental Medicine Biotechnology, College of Life Sciences, Kyung Hee University, Yongin 17104, Republic of Korea

^5^ Natural Product Informatics Research Center, KIST Gangneung Institute of Natural Products, Gangneung 25451, Republic of Korea

**Corresponding author:*

Tel: +82-33-650-3640, Fax: +82-33-650-3679

Email: [kimsm@kist.re.kr](mailto:kimsm@kist.re.kr) (Sang Min Kim)

**Supplementary Tables**

**Table S1**. BLASTN (*megablast*) hits of the three genes in *Wasabia japonica* transcript sequences.

| **A^*^** | **B^*^** | **C^*^** | **D^*^** | **E^*^** | **F^*^** | **G^*^** | **H^*^** | **I^*^** | **J^*^** | **K^*^** | **L^*^** |
| --- | --- | --- | --- | --- | --- | --- | --- | --- | --- | --- | --- |
| *WjMYRI-1* | augustus_masked-ctg477-processed-gene-1.6-mRNA-1 | 94.175 | 206 | 12 | 0 | 798 | 1003 | 102 | 307 | 7.74E-85 | 315 |
| *WjMYRI-1* | augustus_masked-ctg477-processed-gene-1.6-mRNA-1 | 86.973 | 261 | 32 | 2 | 1130 | 1389 | 398 | 657 | 3.63E-78 | 292 |
| *WjMYRI-1* | augustus_masked-ctg477-processed-gene-1.6-mRNA-1 | 96.429 | 84 | 2 | 1 | 298 | 380 | 16 | 99 | 1.80E-31 | 137 |
| *WjMYRI-2* | augustus_masked-ctg477-processed-gene-1.6-mRNA-1 | 84.211 | 209 | 30 | 2 | 796 | 1001 | 103 | 311 | 2.23E-50 | 200 |

^*^ The BLASTN output was prepared in the tabular format (outfmt = 6). **A**: query sequence, **B**: subject sequence, **C**: percentage of identical matches, **D**: alignment length, **E**: the number of mismatches, **F**: the number of gap openings, **G**/**H**: start/end position of an alignment in query, **I**/**J**: start/end position of an alignment in subject, **K**: E-value, and **L**: bit score.

**Table S2**. BLASTN (*blastn*) hits of the three genes in *Wasabia japonica* transcript sequences.

| **A^*^** | **B^*^** | **C^*^** | **D^*^** | **E^*^** | **F^*^** | **G^*^** | **H^*^** | **I^*^** | **J^*^** | **K^*^** | **L^*^** |
| --- | --- | --- | --- | --- | --- | --- | --- | --- | --- | --- | --- |
| *WjMYRI-1* | augustus_masked-ctg477-processed-gene-1.6-mRNA-1 | 81.682 | 333 | 40 | 1 | 1078 | 1389 | 325 | 657 | 1.91E-92 | 341 |
| *WjMYRI-1* | augustus_masked-ctg477-processed-gene-1.6-mRNA-1 | 91.892 | 222 | 18 | 0 | 785 | 1006 | 89 | 310 | 1.79E-86 | 320 |
| *WjMYRI-1* | augustus_masked-ctg477-processed-gene-1.6-mRNA-1 | 96.386 | 83 | 3 | 0 | 298 | 380 | 16 | 98 | 4.22E-31 | 137 |
| *WjMYRI-1* | maker-ctg36-augustus-gene-5.60-mRNA-1 | 73.333 | 120 | 32 | 0 | 465 | 584 | 1152 | 1271 | 4.22E-12 | 73.4 |
| *WjMYRI-1* | maker-ctg36-augustus-gene-5.59-mRNA-1 | 77.333 | 75 | 17 | 0 | 465 | 539 | 414 | 488 | 9.29E-08 | 59.9 |
| *WjMYRI-2* | augustus_masked-ctg477-processed-gene-1.6-mRNA-1 | 83.732 | 209 | 31 | 1 | 796 | 1001 | 103 | 311 | 3.64E-57 | 223 |
| *WjMYRI-2* | augustus_masked-ctg477-processed-gene-1.6-mRNA-1 | 77.083 | 192 | 41 | 2 | 1171 | 1362 | 469 | 657 | 9.78E-33 | 142 |
| *WjMYRI-2* | augustus_masked-ctg477-processed-gene-1.6-mRNA-1 | 81.928 | 83 | 15 | 0 | 292 | 374 | 16 | 98 | 8.04E-15 | 83.3 |
| *WjMYRI-2* | maker-ctg6375-augustus-gene-0.0-mRNA-1 | 71.186 | 118 | 32 | 2 | 492 | 608 | 318 | 434 | 3.90E-06 | 53.6 |
| *WjMYRII* | maker-ctg375-augustus-gene-1.18-mRNA-1 | 70.886 | 158 | 44 | 2 | 490 | 646 | 445 | 601 | 1.39E-11 | 71.6 |
| *WjMYRII* | maker-ctg36-augustus-gene-5.60-mRNA-1 | 74.775 | 111 | 24 | 2 | 434 | 542 | 1097 | 1205 | 4.86E-11 | 69.8 |
| *WjMYRII* | maker-ctg36-augustus-gene-5.59-mRNA-1 | 75.000 | 112 | 26 | 2 | 434 | 544 | 359 | 469 | 4.86E-11 | 69.8 |
| *WjMYRII* | augustus_masked-ctg498-processed-gene-2.13-mRNA-1 | 64.491 | 383 | 123 | 5 | 174 | 545 | 120 | 500 | 1.70E-10 | 68.0 |

^*^ The BLASTN output was prepared in the tabular format (outfmt = 6). **A**: query sequence, **B**: subject sequence, **C**: percentage of identical matches, **D**: alignment length, **E**: the number of mismatches, **F**: the number of gap openings, **G**/**H**: start/end position of an alignment in query, **I**/**J**: start/end position of an alignment in subject, **K**: E-value, and **L**: bit score.

**Table S3**. BLASTP results for the protein sequences of the six non-redundant transcript hits against *Wasabia japonica* protein sequences.

| **A^*^** | **B^*^** | **C^*^** | **D^*^** | **E^*^** | **F^*^** | **G^*^** | **H^*^** | **I^*^** | **J^*^** | **K^*^** | **L^*^** |
| --- | --- | --- | --- | --- | --- | --- | --- | --- | --- | --- | --- |
| WjMYRI-1 | augustus_masked-ctg477-processed-gene-1.6-mRNA-1 | 70.192 | 208 | 36 | 2 | 263 | 463 | 31 | 219 | 1.14E-96 | 292 |
| WjMYRI-1 | augustus_masked-ctg477-processed-gene-1.6-mRNA-1 | 87.500 | 32 | 4 | 0 | 100 | 131 | 6 | 37 | 4.58E-11 | 62.4 |
| WjMYRI-1 | augustus_masked-ctg498-processed-gene-2.13-mRNA-1 | 43.956 | 182 | 93 | 3 | 12 | 190 | 8 | 183 | 1.30E-47 | 163 |
| WjMYRI-1 | maker-ctg36-augustus-gene-5.59-mRNA-1 | 39.744 | 156 | 75 | 5 | 31 | 180 | 21 | 163 | 3.94E-29 | 112 |
| WjMYRI-1 | maker-ctg36-augustus-gene-5.60-mRNA-1 | 35.281 | 445 | 213 | 10 | 31 | 463 | 255 | 636 | 1.40E-78 | 259 |
| WjMYRI-1 | maker-ctg36-augustus-gene-5.60-mRNA-1 | 34.167 | 240 | 131 | 8 | 267 | 501 | 7 | 224 | 5.52E-33 | 133 |
| WjMYRI-1 | maker-ctg375-augustus-gene-1.18-mRNA-1 | 37.500 | 264 | 136 | 4 | 43 | 303 | 18 | 255 | 2.94E-55 | 191 |
| WjMYRI-1 | maker-ctg375-augustus-gene-1.18-mRNA-1 | 41.667 | 48 | 28 | 0 | 444 | 491 | 302 | 349 | 1.05E-06 | 50.8 |
| WjMYRI-1 | maker-ctg6375-augustus-gene-0.0-mRNA-1 | 50.649 | 77 | 38 | 0 | 152 | 228 | 92 | 168 | 1.21E-20 | 91.7 |
| WjMYRI-2 | augustus_masked-ctg477-processed-gene-1.6-mRNA-1 | 50.971 | 206 | 67 | 7 | 264 | 454 | 33 | 219 | 7.46E-55 | 184 |
| WjMYRI-2 | augustus_masked-ctg477-processed-gene-1.6-mRNA-1 | 66.667 | 36 | 12 | 0 | 94 | 129 | 2 | 37 | 7.32E-08 | 52.8 |
| WjMYRI-2 | augustus_masked-ctg498-processed-gene-2.13-mRNA-1 | 44.974 | 189 | 95 | 3 | 3 | 188 | 1 | 183 | 1.05E-49 | 169 |
| WjMYRI-2 | maker-ctg36-augustus-gene-5.59-mRNA-1 | 38.462 | 156 | 77 | 4 | 29 | 178 | 21 | 163 | 9.40E-29 | 111 |
| WjMYRI-2 | maker-ctg36-augustus-gene-5.60-mRNA-1 | 36.175 | 434 | 217 | 9 | 29 | 454 | 255 | 636 | 2.70E-80 | 263 |
| WjMYRI-2 | maker-ctg36-augustus-gene-5.60-mRNA-1 | 33.617 | 235 | 137 | 6 | 263 | 492 | 4 | 224 | 9.41E-33 | 132 |
| WjMYRI-2 | maker-ctg375-augustus-gene-1.18-mRNA-1 | 35.581 | 267 | 141 | 5 | 39 | 301 | 16 | 255 | 2.99E-50 | 177 |
| WjMYRI-2 | maker-ctg375-augustus-gene-1.18-mRNA-1 | 33.708 | 89 | 54 | 2 | 435 | 522 | 302 | 386 | 2.12E-07 | 52.8 |
| WjMYRI-2 | maker-ctg6375-augustus-gene-0.0-mRNA-1 | 56.000 | 75 | 33 | 0 | 150 | 224 | 92 | 166 | 1.98E-23 | 99.4 |
| WjMYRII | augustus_masked-ctg477-processed-gene-1.6-mRNA-1 | 34.762 | 210 | 103 | 5 | 265 | 461 | 31 | 219 | 4.65E-27 | 108 |
| WjMYRII | augustus_masked-ctg498-processed-gene-2.13-mRNA-1 | 58.385 | 161 | 64 | 1 | 41 | 198 | 23 | 183 | 5.67E-61 | 198 |
| WjMYRII | maker-ctg36-augustus-gene-5.59-mRNA-1 | 37.097 | 186 | 77 | 6 | 10 | 188 | 11 | 163 | 4.93E-31 | 117 |
| WjMYRII | maker-ctg36-augustus-gene-5.60-mRNA-1 | 37.069 | 464 | 208 | 12 | 10 | 461 | 245 | 636 | 8.71E-87 | 280 |
| WjMYRII | maker-ctg36-augustus-gene-5.60-mRNA-1 | 36.638 | 232 | 132 | 4 | 269 | 497 | 4 | 223 | 1.30E-35 | 140 |
| WjMYRII | maker-ctg375-augustus-gene-1.18-mRNA-1 | 39.630 | 270 | 136 | 5 | 44 | 310 | 10 | 255 | 9.55E-63 | 210 |
| WjMYRII | maker-ctg6375-augustus-gene-0.0-mRNA-1 | 48.684 | 76 | 39 | 0 | 159 | 234 | 91 | 166 | 8.93E-20 | 89 |

^*^ The BLASTP output was prepared in the tabular format (outfmt = 6). **A**: query sequence, **B**: subject sequence, **C**: percentage of identical matches, **D**: alignment length, **E**: the number of mismatches, **F**: the number of gap openings, **G**/**H**: start/end position of an alignment in query, **I**/**J**: start/end position of an alignment in subject, **K**: E-value, and **L**: bit score.

**Table S4**. Domain profiles for the protein sequences of the three genes and the six non-redundant transcript hits against *Wasabia japonica* protein sequences.

| **Name** | **A^*^** | **B^*^** | **C^*^** | **D^*^** | **E^*^** |
| --- | --- | --- | --- | --- | --- |
| *The three myrosinase genes* |  |  |  |  |  |
| *WjMYRI-1* | 545 | 1 | 1 | 1 | 1 |
| *WjMYRI-2* | 538 | 1 | 1 | 0 | 1 |
| *WjMYRII* | 516 | 1 | 1 | 1 | 1 |
| *The protein sequences of the six non-redundant hits* |  |  |  |  |  |
| augustus_masked-ctg477-processed-gene-1.6-mRNA-1 | 219 | 2 | 1 | 1 | 0 |
| maker-ctg36-augustus-gene-5.60-mRNA-1 | 637 | 2 | 1 | 0 | 1 |
| maker-ctg36-augustus-gene-5.59-mRNA-1 | 163 | 2 | 1 | 0 | 0 |
| maker-ctg6375-augustus-gene-0.0-mRNA-1 | 265 | 2 | 1 | 0 | 0 |
| maker-ctg375-augustus-gene-1.18-mRNA-1 | 404 | 2 | 1 | 0 | 1 |
| augustus_masked-ctg498-processed-gene-2.13-mRNA-1 | 183 | 2 | 1 | 0 | 1 |

* **A**: length in amino acids, and **B-E**: the number of the following domains, IPR001360 (**B**), IPR017853 (**C**), IPR018120 (**D**), and IPR033132 (**E**).

**Table S5.** Content of major glucosinolates found (µmol‧g^-1^) in different organs of *Wasabia japonica* plant. Different superscripted letters indicate significant differences among different analyzed by One-way ANOVA, followed by Tukey’s test, *P*<0.05. Abbreviations: GSL, glucosinolate; n.d., not detected

| **SAMPLES** | **YOUNG LEAF** | **MATURE LEAF** | **YOUNG PETIOLE** | **MATURE PETIOLE** | **ROOT** |
| --- | --- | --- | --- | --- | --- |
| Sinigrin | 73.26 ± 4.87^a^ | 31.70 ± 5.66^b^ | 38.10 ± 1.73^b^ | 32.70 ± 5.10^b^ | 65.08 ± 2.48^a^ |
| Glucohesperin | 4.93 ± 0.26^a^ | 0.44 ± 0.31^e^ | 1.58 ± 0.76^c^ | 0.79 ± 0.25^d^ | 4.23 ± 0.31^b^ |
| Glucoraphasatin | 0.42 ± 0.06^b^ | 0.34 ± 0.16^b^ | 0.67 ± 0.20^a^ | 0.51 ± 0.15^a^ | 0.38 ± 0.03^b^ |
| Glucoibervirin | 4.21 ± 0.55^a^ | 2.30 ± 1.69^c^ | 2.48 ± 0.92^c^ | 3.34 ± 1.16^b^ | 3.76 ± 0.16^b^ |
| (7-methylsulfinyl)heptyl GSL | 2.18 ± 0.09^a^ | 0.22 ± 0.21^c^ | 0.65 ± 0.41^b^ | 0.37 ± 0.26^c^ | 1.91 ± 0.26^a^ |
| Glucoraphenin | 1.09 ± 0.10^a^ | n.d. | 0.90 ± 0.36^a^ | 0.68 ± 0.3^b^ | 1.43 ± 0.11^a^ |
| Glucolesquerellin | 6.77 ± 0.28^a^ | 0.87 ± 0.48^d^ | 5.25 ± 0.99^b^ | 3.72 ± 0.86^c^ | 6.61 ± 0.82^a^ |
| Glucoarabishirutain | 1.546 ± 0.32^a^ | 0.587 ± 0.00^b^ | 1.477 ± 0.77^a^ | 1.122 ± 0.36^a^ | 1.228 ± 0.13^a^ |
| Methoxyglucobrassicin | 0.60 ± 0.06^b^ | 0.14 ± 0.06^c^ | 0.51 ± 0.24^c^ | 0.31 ± 0.12^c^ | 1.03 ± 0.14^a^ |
| Neoglucobrassicin | 1.60 ± 2.43^b^ | n.d | 0.19 ± 0.13^c^ | 0.03 ± 0.03^d^ | 4.27 ± 0.36^a^ |

**Table S6.** Glucosinolate composition and content (µmol‧g^-1^) in different vegetative organs of *Wasabia japonica* plant under abiotic treatments. Asterisks present significant differences between treated group and control group analyzed with Two-tailed t test, * *P*<0.05, ** *P*<0.01, *** *P*<0.001. Abbreviations**:** GSL, glucosinolate; MeJA, methyl jasmonate; n.d., not detected

|  |  | **Abiotic factors** | | | | |
| --- | --- | --- | --- | --- | --- | --- |
|  |  | **Control** | **Drought** | **Salt** | **Salicylic acid** | **MeJA** |
| **LEAF** | Sinigrin | 37.12 ± 0.86 | 27.04 ± 2.84^**^ | 31.34 ± 1.45^**^ | 32.35 ± 0.65^**^ | 29.30 ± 2.12^**^ |
|  | Glucohesperin | 0.36 ± 0.05 | 0.28 ± 0.00 | 0.36 ± 0.03 | 0.38 ± 0.03 | 0.33 ± 0.03 |
|  | Glucoraphasatin | n.d. | n.d. | n.d. | n.d. | n.d. |
|  | Glucoibervirin | 7.13 ± 0.30 | 7.04 ± 0.26 | 8.42 ± 0.92 | 9.55 ± 0.82^**^ | 10.46 ± 0.48^***^ |
|  | 7-(methylsulfinyl)heptyl GSL | n.d. | n.d. | n.d. | n.d. | n.d. |
|  | Glucoraphenin | n.d. | n.d. | 0.36 ± 0.07^***^ | 0.16 ± 0.02^***^ | n.d. |
|  | Glucolesquerellin | 0.75 ± 0.01 | 0.68 ± 0.06 | 1.39 ± 0.21^**^ | 1.00 ± 0.03^***^ | 0.90 ± 0.20 |
|  | Glucoarabishirutain | n.d. | 0.20 ± 0.03^***^ | 0.34 ± 0.15^***^ | 0.26 ± 0.03^***^ | 0.25 ± 0.07^***^ |
|  | Methoxyglucobrassicin | 0.24 ± 0.02 | 0.28 ± 0.01^*^ | 0.30 ± 0.04 | 0.24 ± 0.01 | 0.31 ± 0.04 |
|  | Neoglucobrassicin | n.d. | n.d. | n.d. | n.d. | n.d. |
| **PETIOLE** | Sinigrin | 38.67 ± 2.21 | 38.78 ± 1.44 | 40.16 ± 0.86 | 39.68 ± 2.57 | 38.43 ± 0.79 |
|  | Glucohesperin | 1.05 ± 0.15 | 0.54 ± 0.03^**^ | 0.86 ± 0.01 | 0.81 ± 0.04 | 0.76 ± 0.04^*^ |
|  | Glucoraphasatin | 0.20 ± 0.01 | 0.21 ± 0.01 | 0.23 ± 0.00^ns^ | 0.23 ± 0.02 | 0.21 ± 0.01 |
|  | Glucoibervirin | 4.15 ± 0.23 | 5.25 ± 0.40^*^ | 7.03 ± 0.13^***^ | 6.09 ± 0.41^**^ | 6.16 ± 0.12^***^ |
|  | 7-(methylsulfinyl)heptyl GSL | 0.60 ± 0.10 | 0.24 ± 0.02^**^ | 0.27 ± 0.01^**^ | 0.39 ± 0.03^*^ | 0.39 ± 0.01^*^ |
|  | Glucoraphenin | 0.43 ± 0.05 | 0.44 ± 0.09 | 1.11 ± 0.01^***^ | 0.54 ± 0.04^*^ | 0.53 ± 0.06 |
|  | Glucolesquerellin | 3.36 ± 0.31 | 2.77 ± 0.11^*^ | 4.43 ± 0.07^**^ | 3.80 ± 0.32 | 3.76 ± 0.21 |
|  | Glucoarabishirutain | 0.96 ± 0.07 | 0.79 ± 0.09 | 0.98 ± 0.20 | 1.20 ± 0.02^**^ | 1.12 ± 0.20 |
|  | Methoxyglucobrassicin | 0.27 ± 0.01 | 0.23 ± 0.02^*^ | 0.27 ± 0.01 | 0.18 ± 0.01^***^ | 0.20 ± 0.07 |
|  | Neoglucobrassicin | n.d. | n.d. | n.d. | n.d. | n.d. |
| **ROOT** | Sinigrin | 70.53 ± 2.92 | 73.21 ± 2.30 | 70.54 ± 3.84 | 71.06 ± 1.90 | 64.63 ± 0.84^**^ |
|  | Glucohesperin | 4.13 ± 0.37 | 2.80 ± 0.08^**^ | 2.07 ± 0.11^**^ | 2.39 ± 0.06^**^ | 2.82 ± 0.09^**^ |
|  | Glucoraphasatin | 0.42 ± 0.02 | 0.51 ± 0.01^*^ | 0.41 ± 0.02 | 0.44 ± 0.00 | 0.39 ± 0.02 |
|  | Glucoibervirin | 3.11 ± 0.17 | 2.61 ± 0.02^**^ | 6.59 ± 0.42^***^ | 5.54 ± 0.07^***^ | 5.65 ± 0.24^***^ |
|  | 7-(methylsulfinyl)heptyl GSL | 1.69 ± 0.29 | 1.12 ± 0.05^*^ | 0.68 ± 0.04^**^ | 0.89 ± 0.03^**^ | 1.02 ± 0.02^*^ |
|  | Glucoraphenin | 1.06 ± 0.06 | 1.04 ± 0.03 | 2.07 ± 0.07^***^ | 1.37 ± 0.05^**^ | 1.49 ± 0.38 |
|  | Glucolesquerellin | 5.28 ± 0.26 | 4.80 ± 0.03^*^ | 8.49 ± 0.31^***^ | 7.52 ± 0.12^***^ | 6.38 ± 0.22^**^ |
|  | Glucoarabishirutain | 1.14 ± 0.47 | 0.79 ± 0.21 | 1.48 ± 0.17 | 1.15 ± 0.10 | 0.90 ± 0.15 |
|  | Methoxyglucobrassicin | 0.94 ± 0.02 | 0.68 ± 0.04^***^ | 1.11 ± 0.03^***^ | 1.60 ± 0.17^**^ | 0.90 ± 0.05 |
|  | Neoglucobrassicin | 6.45 ± 0.28 | 3.14 ± 0.09^***^ | 6.14 ± 0.30 | 6.57 ± 0.21 | 5.64 ± 0.06^**^ |

**Table S7.** Composition and content (µmol‧g^-1^) of glucosinolate hydrolysis products in different organs of *Wasabia* *japonica* plant. Relative amount was determined using standard curves constructed with allyl isothiocyanate and phenyl isothiocyanate. Different alphabets represent significant differences among plant tissues analyzed with one-way ANOVA, followed by Tukey’s test (*P*<0.05). Abbreviations: MeJA, methyl jasmonate; NCS, isothiocyanate; n.d., not detected

| **SAMPLES** | **YOUNG LEAF** | **MATURE LEAF** | **YOUNG PETIOLE** | **MATURE PETIOLE** | **ROOT** |
| --- | --- | --- | --- | --- | --- |
| Isopropyl NCS | 0.06 ± 0.01^b^ | n.d. | 0.06 ± 0.01^b^ | 0.02 ± 0.01^c^ | 0.09 ± 0.02^a^ |
| Allyl thiocyanate | 3.32 ± 0.40^a^ | 0.41 ± 0.06^c^ | 2.18 ± 0.09^b^ | 0.65 ± 0.13^c^ | 3.28 ± 0.24^a^ |
| Allyl NCS | 72.07 ± 7.51^a^ | 14.12 ± 1.29^c^ | 49.28 ± 1.69^b^ | 20.19 ± 3.55^c^ | 69.31 ± 4.89^a^ |
| Isobutyl NCS | 0.39 ± 0.05^a^ | 0.19 ± 0.12^bc^ | 0.28 ± 0.05^ab^ | 0.15 ± 0.03^c^ | 0.37 ± 0.02^a^ |
| Butyl NCS | 0.19 ± 0.08^a^ | n.d. | 0.10 ± 0.08^a^ | n.d. | 0.21 ± 0.09^a^ |
| 3-butenyl NCS | 0.38 ± 0.22^ab^ | n.d. | 0.16 ± 0.07^b^ | n.d. | 0.48 ± 0.07^a^ |
| 4-pentenyl NCS | 0.23 ± 0.04^b^ | n.d. | 0.06 ± 0.03^c^ | n.d. | 0.42 ± 0.01^a^ |
| 5-hexenyl NCS | n.d. | n.d. | n.d. | n.d | 0.67 ± 0.01 |
| Heptenyl NCS | 0.33 ± 0.01^a^ | n.d. | n.d. | n.d. | 0.38 ± 0.01^a^ |
| Iberverin | 0.51 ± 0.05^b^ | n.d | 0.15 ± 0.00^c^ | 0.42 ± 0.18^b^ | 2.74 ± 0.52^a^ |
| Beteroin | n.d. | n.d. | n.d. | n.d. | 0.42 ± 0.08 |
| Unknown | 0.05 ± 0.01^b^ | 0.03 ± 0.01^b^ | 0.04 ± 0.00^b^ | 0.03 ± 0.00^b^ | 0.10 ± 0.02^a^ |
| Lesquerellin | 1.03 ± 0.19^b^ | n.d. | 0.24 ± 0.04^c^ | 0.34 ± 0.02^c^ | 2.40 ± 0.31^a^ |
| 1-(methyl)indole-2-carboxylic acid | n.d. | n.d. | n.d. | n.d. | 0.07 ± 0.05 |
| 7-methylthioheptyl NCS | 0.72 ± 0.15^a^ | n.d. | 0.32 ± 0.04^b^ | n.d. | 0.28 ± 0.05^b^ |
| Hesperin | 1.42 ± 0.01^b^ | n.d. | 0.49 ± 0.24^c^ | n.d. | 2.50 ± 0.03^a^ |

**Table S8.** Composition and content (µmol‧g^-1^) of glucosinolate hydrolysis products in different vegetative organs of wasabi plant under abiotic treatments. Relative amount was determined using standard curves constructed with allyl isothiocyanate and phenyl isothiocyanate. Asterisks present significant differences between treated group and control group analyzed with Two-tailed t test, * *P*<0.05, ** *P*<0.01, *** *P*<0.001. Abbreviations: MeJA, methyl jasmonate; NCS, isothiocyanate; n.d., not detected

|  |  | **Abiotic factors** | | | | |
| --- | --- | --- | --- | --- | --- | --- |
|  |  | **Control** | **Drought** | **Salt** | **Salicylic acid** | **MeJA** |
| **LEAF** | Isopropyl NCS | 0.02 ± 0.00 | 0.01 ± 0.00 | 0.04 ± 0.00^*^ | 0.02 ± 0.00 | 0.02 ± 0.00 |
|  | Allyl thiocyanate | 1.06 ± 0.08 | 0.49 ± 0.02^*^ | 1.36 ± 0.21 | 1.06 ± 0.14 | 0.98 ± 0.04 |
|  | Allyl NCS | 23.33 ± 1.52 | 14.51 ± 0.32^*^ | 32.64 ± 4.19 | 26.92 ± 2.68 | 24.36 ± 0.62 |
|  | Isobutyl NCS | 0.06 ± 0.00 | 0.08 ± 0.03 | 0.12 ± 0.02^*^ | 0.08 ± 0.00^***^ | 0.08 ± 0.00^**^ |
|  | Butyl NCS | n.d. | n.d. | n.d. | 0.06 ± 0.04 | 0.07 ± 0.04 |
|  | 3-butenyl NCS | 0.03 ± 0.00 | 0.01 ± 0.01 | 0.09 ± 0.02 | 0.05 ± 0.03 | 0.13 ± 0.01^**^ |
|  | 4-pentenyl NCS | n.d. | n.d. | n.d. | n.d. | n.d. |
|  | 5-hexenyl NCS | n.d. | n.d. | n.d. | n.d. | n.d. |
|  | Heptenyl NCS | n.d. | n.d. | n.d. | n.d. | n.d. |
|  | Iberverin | 0.30 ± 0.04 | 0.91 ± 0.10^*^ | 2.27 ± 0.66 | 1.03 ± 0.27 | 1.14 ± 0.17^*^ |
|  | Beteroin | n.d. | n.d. | n.d. | n.d. | n.d. |
|  | Unknown | 0.05 ± 0.00 | 0.04 ± 0.01 | 0.06 ± 0.03 | 0.03 ± 0.00^*^ | 0.02 ± 0.00^*^ |
|  | Lesquerellin | n.d. | n.d. | n.d. | n.d. | n.d. |
|  | 1-(methyl)indole-2-carboxylic acid | n.d. | n.d. | n.d. | n.d. | n.d. |
|  | 7-methylthioheptyl NCS | n.d. | n.d. | n.d. | n.d. | n.d. |
|  | Hesperin | n.d. | n.d. | n.d. | n.d. | n.d. |
| **PETIOLE** | Isopropyl NCS | n.d. | 0.04 ± 0.00 | 0.09 ± 0.01 | 0.04 ± 0.01 | 0.05 ± 0.00 |
|  | Allyl thiocyanate | 0.47 ± 0.08 | 1.05 ± 0.01^*^ | 1.94 ± 0.12^**^ | 1.63 ± 0.19^*^ | 1.30 ± 0.11^*^ |
|  | Allyl NCS | 13.68 ± 0.38 | 29.20 ± 0.15^***^ | 44.95 ± 2.27^**^ | 38.36 ± 4.04^*^ | 31.81 ± 2.19^**^ |
|  | Isobutyl NCS | 0.42 ± 0.52 | 0.12 ± 0.00^*^ | 0.29 ± 0.01 | 0.19 ± 0.01 | 0.12 ± 0.01 |
|  | Butyl NCS | n.d. | 0.15 ± 0.01 | 0.10 ± 0.00 | 0.07 ± 0.01 | n.d. |
|  | 3-butenyl NCS | n.d. | 0.02 ± 0.01 | 0.13 ± 0.02 | 0.12 ± 0.01 | 0.09 ± 0.01 |
|  | 4-pentenyl NCS | n.d. | n.d. | 0.06 ± 0.01 | n.d. | 0.02 ± 0.00 |
|  | 5-hexenyl NCS | n.d. | n.d. | n.d. | n.d. | n.d. |
|  | Heptenyl NCS | 0.07 ± 0.00 | n.d. | 0.06 ± 0.02 | 0.08 ± 0.01 | 0.06 ± 0.00 |
|  | Iberverin | 0.49 ± 0.07 | 1.23 ± 0.05^**^ | 4.42 ± 0.17^**^ | 2.85 ± 0.44^*^ | 2.31 ± 0.03^***^ |
|  | Beteroin | n.d. | n.d. | 0.18 ± 0.05 | n.d. | n.d. |
|  | Unknown | 0.03 ± 0.01 | 0.07 ± 0.04 | 0.05 ± 0.00 | 0.03 ± 0.01 | 0.03 ± 0.01 |
|  | Lesquerellin | 0.31 ± 0.18 | 0.39 ± 0.01 | 2.06 ± 0.01^**^ | 1.30 ± 0.38 | 0.92 ± 0.03^*^ |
|  | 1-(methyl)indole-2-carboxylic acid | n.d. | n.d. | n.d. | n.d. | n.d. |
|  | 7-methylthioheptyl NCS | 0.32 ± 0.14 | 0.19 ± 0.00 | 0.55 ± 0.04 | 0.68 ± 0.13 | 0.47 ± 0.00 |
|  | Hesperin | 0.15 ± 0.13 | n.d. | 0.14 ± 0.03 | 0.12 ± 0.04 | 0.09 ± 0.01 |
| **ROOT** | Isopropyl NCS | 0.05 ± 0.01 | 0.10 ± 0.01^*^ | 0.11 ± 0.01^*^ | 0.05 ± 0.00 | 0.09 ± 0.01^*^ |
|  | Allyl thiocyanate | 2.43 ± 0.19 | 4.02 ± 0.01^**^ | 3.86 ± 0.21^*^ | 3.45 ± 0.07^*^ | 3.60 ± 0.14^*^ |
|  | Allyl NCS | 52.82 ± 4.03 | 93.61 ± 10.12^*^ | 82.52 ± 3.80^*^ | 73.89 ± 1.87^*^ | 75.99 ± 3.47^*^ |
|  | Isobutyl NCS | 0.19 ± 0.03 | 0.45 ± 0.10 | 0.41 ± 0.01^**^ | 0.34 ± 0.03^*^ | 0.38 ± 0.03^*^ |
|  | Butyl NCS | 0.06 ± 0.02 | 0.16 ± 0.10 | 0.15 ± 0.00 | 0.12 ± 0.02 | 0.12 ± 0.03 |
|  | 3-butenyl NCS | 0.36 ± 0.02 | 0.69 ± 0.03^**^ | 0.41 ± 0.12 | 0.45 ± 0.04 | 0.46 ± 0.13 |
|  | 4-pentenyl NCS | 0.29 ± 0.02 | 0.38 ± 0.02^*^ | 0.43 ± 0.10 | 0.26 ± 0.11 | 0.41 ± 0.01^*^ |
|  | 5-hexenyl NCS | 0.49 ± 0.01 | 0.45 ± 0.04 | 0.45 ± 0.03 | 0.39 ± 0.03^*^ | 0.58 ± 0.02^*^ |
|  | Heptenyl NCS | 0.28 ± 0.00 | 0.33 ± 0.09^***^ | 0.23 ± 0.03 | 0.22 ± 0.01 | 0.28 ± 0.01^*^ |
|  | Iberverin | 1.24 ± 0.09 | 1.28 ± 0.26 | 3.48 ± 0.37^*^ | 2.97 ± 0.21^**^ | 3.40 ± 0.13^**^ |
|  | Beteroin | 0.21 ± 0.02 | 0.27 ± 0.03^*^ | 0.96 ± 0.17 | 0.55 ± 0.08 | 0.54 ± 0.04^*^ |
|  | Unknown | 0.05 ± 0.01 | 0.08 ± 0.00 | 0.06 ± 0.02 | 0.05 ± 0.01 | 0.08 ± 0.00 |
|  | Lesquerellin | 1.37 ± 0.25 | 1.28 ± 0.13 | 3.74 ± 0.61^*^ | 3.16 ± 0.51^*^ | 3.23 ± 0.26 |
|  | 1-(methyl)indole-2-carboxylic acid | 0.06 ± 0.03 | 0.14 ± 0.01 | 0.27 ± 0.06^*^ | 0.29 ± 0.04^*^ | 0.15 ± 0.02 |
|  | 7-methylthioheptyl NCS | 0.18 ± 0.02 | 1.28 ± 0.09^**^ | 0.52 ± 0.10 | 0.45 ± 0.06^*^ | 0.32 ± 0.02 |
|  | Hesperin | 2.25 ± 0.21 | 1.93 ± 0.05 | 1.71 ± 0.27 | 1.45 ± 0.04 | 2.72 ± 0.03 |

| **Variables** | ***WjMYRI-1*** | ***WjMYRII*** | **Sinigrin** | **Glucoibervirin** | **Glucohesperin** | **Glucolesquerellin** |
| --- | --- | --- | --- | --- | --- | --- |
| *WjMYRI-1* |  |  |  |  |  |  |
| *WjMYRII* | 0.0627 |  |  |  |  |  |
| Sinigrin | **0.739** | -0.184 |  |  |  |  |
| Allyl thiocyanate | **0.690** | -0.141 | **0.912** |  |  |  |
| Allyl NCS | **0.685** | -0.135 | **0.903** |  |  |  |
| Glucoibervirin | -0.036 | 0.269 |  |  |  |  |
| Ibervirin | 0.255 | 0.122 |  | 0.322 |  |  |
| Glucohesperin | 0.441 | -0.215 |  |  |  |  |
| Hesperin | 0.569 | -0.217 |  |  | **0.870** |  |
| Glucolesquerellin | 0.544 | -0.122 |  |  |  |  |
| Lesquerellin | **0.612** | -0.103 |  |  |  | **0.838** |

**Table S9. Correlation between glucosinolate, hydrolytic product content, and transcript expression of *WjMYRI-1* and *WjMYRII* genes.** The correlation efficiency was determined with Pearson correlation analysis. Bold numbers denote strong correlation between two factors

**Table S10.** Primer sequences used in quantitative real-time polymerase chain reaction to determine relative mRNA expression of identified myrosinase isogenes in *Wasabia* *japonica.* Abbreviations: Fw, forward; Rv, reverse

| **Gene ID** |  | **OligoNu sequence (5’-3’)** | **Melting temperature** | **Amplicon size** |
| --- | --- | --- | --- | --- |
| *WjTUB-β6* | Fw | CCACTCCTAGCTTTGGTGAT | 55.9 ℃ | 173 bp |
|  | Rv | GGAGGTGAGAGGTGCAAAAC | 57.8 ℃ |  |
| *WjMYRI-1* | Fw | CTTCCCAACTTCACGGAAGCAGAAG | 63 ℃ | 140 bp |
|  | Rv | GCGTCCATCATGGCAGTGTGA | 62.3 ℃ |  |
| *WjMYRI-2* | Fw | GGAGAAGGGTCGTATCCACC | 59.25 ℃ | 103 bp |
|  | Rv | CGTGAGCTAGGAGTTGGTGA | 59.11 ℃ |  |
| *WjMYRII* | Fw | ACGTTACAACCCGACTCCTT | 58.96 ℃ | 124 bp |
|  | Rv | GATATAGTAGCGCCGGTGGT | 59.11 ℃ |  |


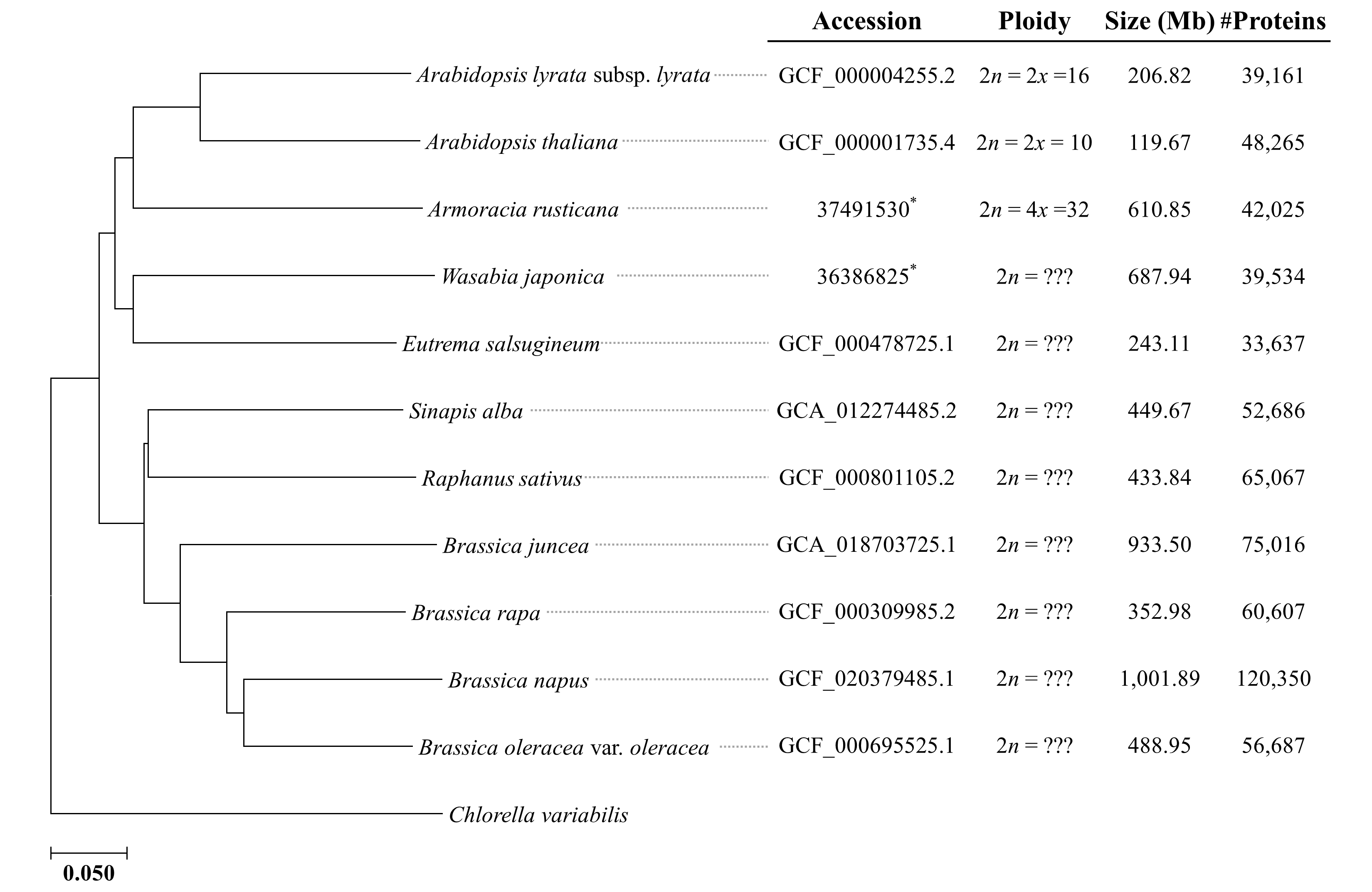


**Figure S1. A phylogenomic tree depicting the genetic evolution of *Wasabia* *japonica* M. within the cruciferous family.** A phylogenomic tree was constructed to show evolutionary relationships among the eleven species featured in Figure 1. The tree is supplemented with information on proteome data sources, ploidy, genome size, and the number of predicted proteins for each species. ^*^PMID for the source publication, if data were no available from NCBI GenBank/RefSeq. Question marks denote for unknown information.


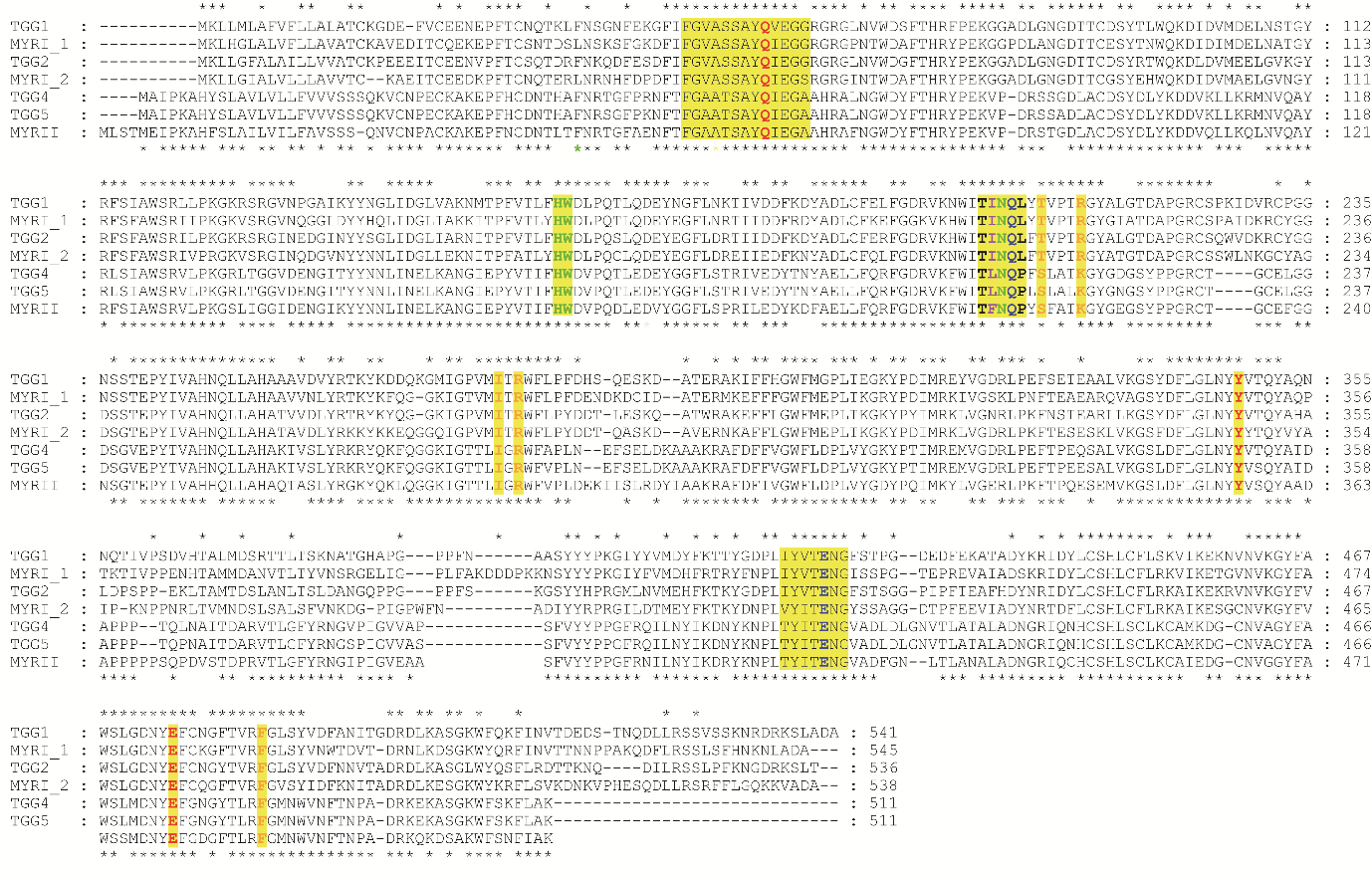
**Figure S2. Multiple sequence alignment of identified myrosinase isogenes in *Wasabia* *japonica* and *Arabidopsis* *thaliana* using ClustalW online tool.** Blue letters are residues of acid-base and catalytic nucleophile. Green letters are specific substrate binding amino acids in myrosinase. Purple letters are substrate recognition amino acids in *ß*-glucosidase family. Red letters are substrate binding amino acids in myrosinase. Orange letters are aglycone binding amino acids in myrosinase. Motif 1 and 2 are two conserved motifs found in myrosinase clade I and II. Red box is N-terminal signature in glycosyl hydrolase family 1.
